# Neuroprotective effects of p62(SQSTM1)-engineered lactic acid bacteria in Alzheimer’s disease: a pre-clinical study

**DOI:** 10.18632/aging.103900

**Published:** 2020-08-28

**Authors:** Valentina Cecarini, Laura Bonfili, Olee Gogoi, Solomon Lawrence, Franco M. Venanzi, Vasco Azevedo, Pamela Mancha-Agresti, Mariana Martins Drumond, Giacomo Rossi, Sara Berardi, Livio Galosi, Massimiliano Cuccioloni, Mauro Angeletti, Jan S. Suchodolski, Rachel Pilla, Jonathan A. Lidbury, Anna Maria Eleuteri

**Affiliations:** 1 School of Biosciences and Veterinary Medicine, University of Camerino, Via Gentile III da Varano, Camerino, Italy; 2 CureLab Oncology, Inc., Dedham, Boston, MA 02026, USA; 3 Laboratório de Genética Celular e Molecular, Instituto de Ciências Biológicas, Departamento de Biologia Geral, Universidade Federal de Minas Gerais, Belo Horizonte, Brazil; 4 FAMINAS- BH, Belo Horizonte, Minas Gerais, Brazil; 5 Centro Federal de Educação Tecnológica de Minas Gerais (CEFET/MG), Departamento de Ciências Biológicas, Belo Horizonte, Brazil; 6 Gastrointestinal Laboratory, Department of Small Animal Clinical Science, Texas A&M University, College Station, TX 77843, USA

**Keywords:** Alzheimer’s disease, p62, engineered lactic acid bacteria, microbiota, inflammation

## Abstract

Alzheimer’s disease (AD) is a progressive neurodegeneration characterized by neuron death ending in memory and cognitive decline. A major concern in AD research is the identification of new therapeutics that could prevent or delay the onset of the disorder, with current treatments being effective only in reducing symptoms. In this perspective, the use of engineered probiotics as therapeutic tools for the delivery of molecules to eukaryotic cells is finding application in several disorders. This work introduces a new strategy for AD treatment based on the use of a *Lactobacillus* *lactis* strain carrying one plasmid (pExu) that contains a eukaryotic expression cassette encoding the human p62 protein. 3xTg-AD mice orally administered with these bacteria for two months showed an increased expression of endogenous p62 in the brain, with a protein delivery mechanism involving both lymphatic vessels and neural terminations, and positive effects on the major AD hallmarks. Mice showed ameliorated memory, modulation of the ubiquitin-proteasome system and autophagy, reduced levels of amyloid peptides, and diminished neuronal oxidative and inflammatory processes. Globally, we demonstrate that these extremely safe, non-pathogenic and non-invasive bacteria used as delivery vehicles for the p62 protein represent an innovative and realistic therapeutic approach in AD.

## INTRODUCTION

Alzheimer’s disease (AD) is an age-related progressive neurodegenerative condition and the most common cause of dementia. AD is characterized by neuronal cell loss associated with memory and cognitive decline. It mainly affects the hippocampus, the entorhinal and the cerebral cortex. Extracellular aggregates of insoluble amyloid-β (Aβ) protein [1, 2], intracellular neurofibrillary tangles of the microtubule-associated protein tau [3], deficit in neurotransmitters and mitochondrial dysfunctions are characteristic markers of this pathology. Additionally, other harmful factors common to all neurodegenerative conditions act as major contributors to AD, among these, the decreased functionality of proteolytic systems, inflammatory processes with increased release of pro-inflammatory cytokines and oxidative damage to cellular macromolecules. Treatments currently approved by the US Food and Drug Administration include drugs that are selectively used against a specific target to delay the progression of symptoms associated with AD. Research efforts are now directed towards the discovery of new disease-modifying therapies, which can block the progression of the disease and affect multiple molecular pathways [4].

Oral bacteriotherapy with probiotics is considered a successful approach for the treatment of different pathologies [5–8]. Furthermore, oral drug delivery is certainly a preferred method due to patient acceptance and rare side effects. Recently, the use of genetically modified and plasmid-containing bacteria as delivery vehicles of therapeutic molecules such as antigens or cytokines to eukaryotic cells is gaining increasing attention. These innovative strategies are mainly based on the use of safe, food-grade bacteria like lactic acid bacteria [9–13]. In particular, *Lactobacillus lactis* is a well characterized model for plasmid delivery to epithelial cell membranes and is intensively studied because, besides its safety, is also considered as a transient, non-colonizing and non-invasive bacterium [14].

This study aims to explore the neuroprotective effects of the *L. lactis* MG1363 strain harboring the newly constructed pExu plasmid containing a eukaryotic expression cassette designed to express the human SQSTM1/p62 (sequestosome 1) (hereafter p62). p62 is a multifunctional protein of 440 amino acids, which contains several structural domains that give it scaffolding ability [15]. It behaves like a signaling hub for multiple pathways including protein turnover via the ubiquitin-proteasome system (UPS) and autophagy, cell proliferation and death, oxidative stress, inflammation and immune response [16]. Previous data described p62 as a common component of protein inclusions that damage brain regions in various neurodegenerative conditions, among them neurofibrillary tangles in AD [17]. In addition, Du et al. demonstrated the occurrence of oxidative damage to the p62 promoter that correlated with decreased expression of p62 in the brain of transgenic AD mice [18, 19]. Recently, Caccamo et al. suggested that increasing p62 levels through a gene therapy strategy can facilitate amyloid-β removal by autophagy activation [20]. In this work, 3xTg-AD mice in the early stage of the pathology were orally administered with p62-engineered Lactobacilli for a two-month period and the effects on AD main neurological hallmarks were investigated. In addition, changes in p62 levels together with the protein distribution in mice tissues and the possible involvement of the gut-brain axis were dissected. The overall result of mice oral treatment was the up-regulation of endogenous p62, associated with improved memory function, modulation of neuronal proteolysis, and decreased AD typical signs including Aβ pathology, inflammatory and oxidative processes.

## RESULTS

### Evaluation of treatment-related toxicity

3xTg-AD mice received a daily oral administration of 10^9^ CFU live p62-engineered LAB or corresponding controls for a period of two months. The treatment did not cause mortality or toxicity signs in any of the animals. No diarrhea, changes in general appearance or other treatment-related sickness were recorded and there was no weight loss or reduction in food intake (data not shown).

### Effects of p62-LAB treatment on mice cognitive functions

**Figure 1:**
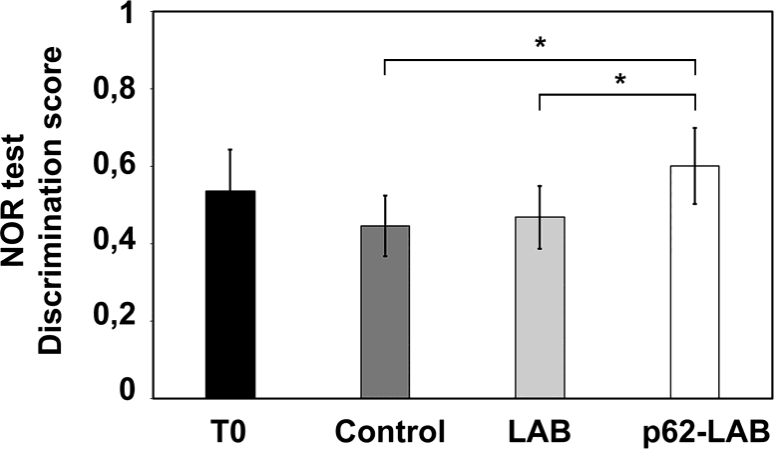
**Novel Object Recognition (NOR) test.** The effect of p62-LAB treatment on mice memory performance was assessed by the NOR test. The discrimination scores obtained for the four groups of mice are reported. The asterisk indicates statistical significance (p<0.05).

Prior to sacrifice, the novel object recognition (NOR) test was performed on the four groups of mice to evaluate the effects of the treatment on hippocampal functions and recognition memory. No significant difference was observed comparing control, LAB and p62-LAB groups with the T0 group whereas a statistically significant difference (p<0.05) was observed in p62-LAB treated mice with respect to both control and LAB group, as shown in Figure 1. In detail, AD mice treated with p62-LAB showed a better “discrimination index” than control and LAB treated animals of the same age.

### Functionality of the LAB:pExu vector and distribution of the p62 protein in mice tissues

The functionality of the LAB-pExu system was evaluated upon the oral administration of *L. lactis* MG1363 (pExu:*egfp*) to 3xTg-AD mice (see Supplementary Materials and Methods). Interestingly, the expression of the eGFP was detected in intestinal cells 6 h after the treatment and in brain cells from 48 h to 72 h after gavage (see Supplementary Materials and Methods and Supplementary Figure 1).

**Figure 2:**
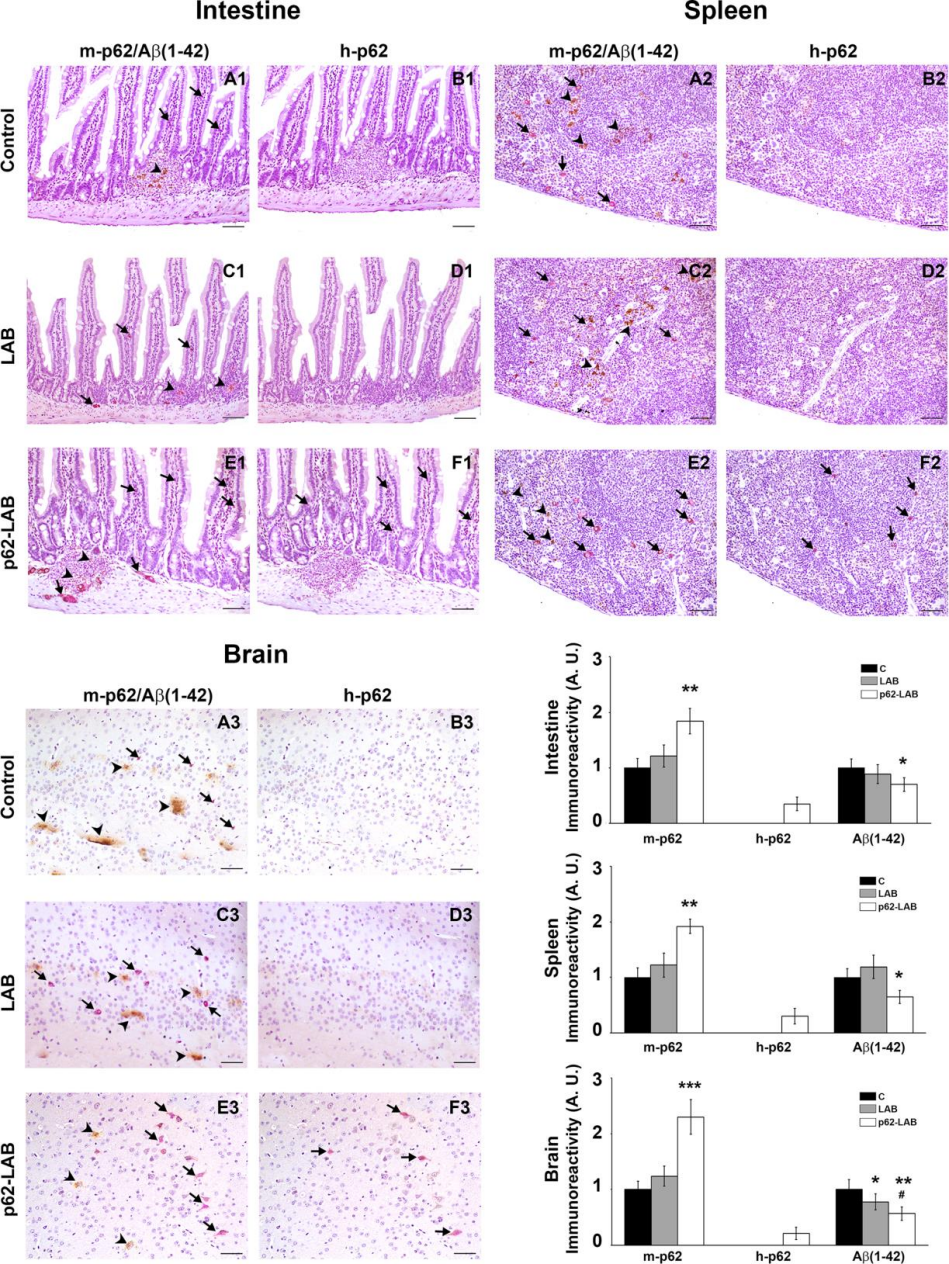
**IHC analysis of intestine, spleen and brain from control, LAB and p62-LAB treated mice.** Tissue sections from duodenal mucosa, spleen and brain, specifically the hippocampal region, were stained with an anti-rodent p62 antibody (purple), an anti-human p62 antibody (purple) and an anti-Aβ(1-42) antibody (brown). Panels **A1**-**B1**, **C1**-**D1**, and **E1**-**F1** show consecutive intestinal sections, panels **A2**-**B2**, **C2**-**D2**, and **E2**-**F2** show consecutive spleen sections and panels **A3**-**B3**, **C3**-**D3**, and **E3**-**F3** show consecutive brain sections. Immunohistochemical double-staining for m-p62 (arrows) and Aβ(1-42) (arrowheads) is shown (IHC, intestine and spleen 20×, scale bar = 100 μm; brain 40×, scale bar = 200 μm, ^*^p<0.05, ^**^p<0.01 vs C and LAB, ^#^p<0.05 vs LAB).

**Figure 3:**
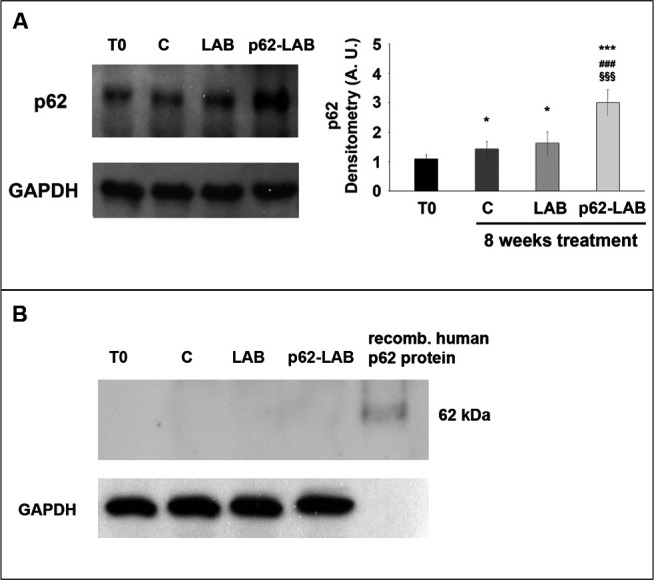
**Immunodetection of the p62 protein in brain homogenates of T0, control and treated 3xTg-AD mice.** Panel **A** shows detection of the m-p62 using a monoclonal anti-murine p62 antibody. GAPDH was used as a control to check equal protein loading. Densitometry is shown on the right (^*^p<0.05 and ^***^p<0.001 vs T0, ^###^p<0.001 vs C, ^§§§^p<0.001 vs LAB). Panel **B** shows detection of the h-p62 protein in brain homogenates of T0, control (**C**) and treated 3xTg-AD mice using a monoclonal anti-human p62 antibody. 3 μg of a recombinant human p62 protein were loaded as control. GAPDH was used as a control to check equal protein loading.

The distribution of the p62 protein, both the endogenous (murine, m-p62) and the exogenous (human and plasmid-associated, h-p62) form, was determined by immunohistochemistry (IHC) of intestinal, lymphoid and nervous tissues from control, LAB and p62-LAB groups (Figure 2). In order to discriminate between the two p62 isoforms, the analysis was conducted on sequential slices using an anti-rodent and an anti-human p62 monoclonal antibody. In untreated and LAB treated mice, the immunohistochemical analysis of duodenal mucosa evidenced endogenous p62 in few scattered mononuclear cells along villi (Figure 2, panels A1 and C1). Interestingly, p62-LAB treatment induced an evident increase of m-p62 in neuronal ganglia and in some mononuclear and interstitial cells of villi (Figure 2, panel E1). A sporadic and weak expression of the exogenous p62 in interstitial cells of the villi was observed (Figure 2, panel F1). In the spleen, a modest positivity for endogenous p62 was measured in control and LAB treated mice (Figure 2, panel A2 and C2) whereas p62-LAB treatment induced a clear expression of the same protein in many mononuclear and megakaryocyte cells of the white pulp (Figure 2, panel E2). The analysis of consecutive spleen sections revealed a weak expression of the h-p62 (Figure 2, panel F2). Finally, in the hippocampal region of control and LAB brains, a poor expression of the endogenous form of p62 was observed (Figure 2, panels A3 and C3). Similar to intestine and spleen, brains of p62-LAB treated mice showed an evident up-regulation of endogenous p62 (Figure 2, panel E3) and a rare positivity for the human p62, with few weakly labeled neurons in the hippocampus, evident only at higher magnification of the neuronal region (Figure 2, panel F3). Concerning the brain of p62-LAB mice, neuronal cells showed a weak-to-strong cytoplasmic reactivity to p62 (the most significant signals are indicated by the arrows). No positivity was observed for h-p62 in tissue sections from control mice and LAB treated mice (Figure 2, panels B1, B2 and B3, D1, D2 and D3). An increased expression of the endogenous p62 protein upon p62-LAB treatment was also detected in mesenteric lymph nodes (Supplementary Figure 2). These *in-situ* morphological data on murine p62 up-regulation was then confirmed by western blot analysis performed on total brain homogenates (Figure 3, panel A). No signal was detected for the human p62 protein (Figure 3, panel B), likely due to the small percentage of neuronal cells expressing this protein.

### Reduction of brain amyloid load after oral administration of p62-LAB

**Figure 4:**
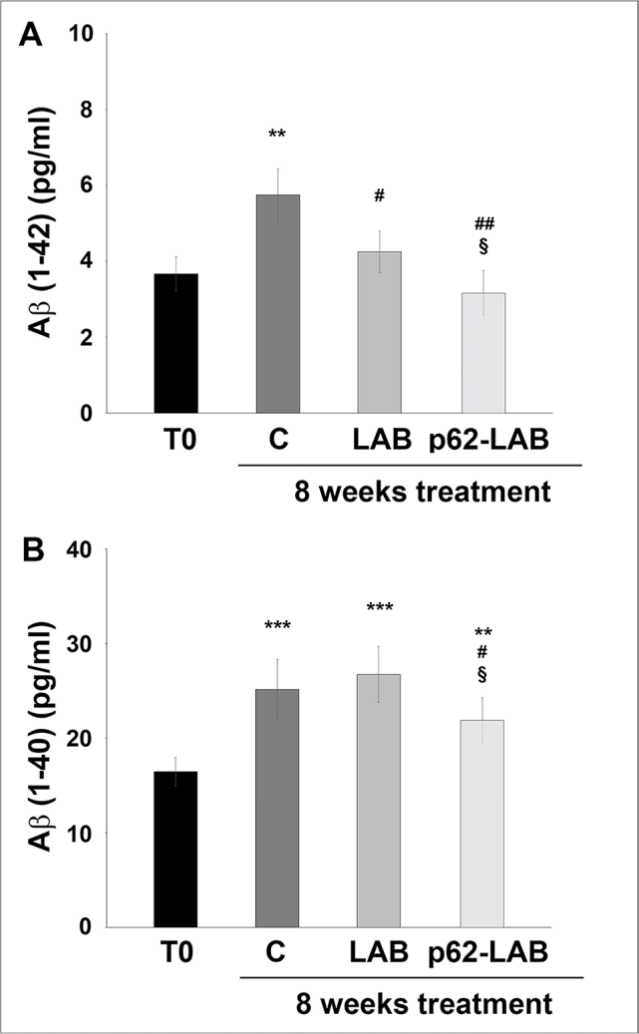
**Amyloid peptides load in the brain of 3xTg-AD mice.** Levels of Aβ(1-42) (panel **A**) and Aβ(1-40) (panel **B**) peptides measured by ELISA on brain homogenates of 3xTg-AD mice, T0, C, LAB and p62-LAB groups. Concentrations are expressed as pg/mL (**p<0.01 and ***p<0.001 vs T0, ^#^p<0.05 and ^##^p<0.01 vs C, ^§^p<0.05 vs LAB).

Increased production and release of amyloid peptides are major hallmarks of AD and responsible for neuronal degeneration. Herein, the effect of p62-LAB treatment on the amyloid load in the brain, spleen and intestine of 3xTg-AD mice was evaluated through IHC and ELISA. Furthermore, in order to establish a correlation between p62 expression and Aβ(1–42) peptide levels, a double staining analysis (purple and brown staining, respectively) was performed in the abovementioned tissues (Figure 2). In detail, aggregates of Aβ(1-42), shown in the sections as brown deposits, were observed in the duodenal mucosa of control and LAB treated animals (Figure 2, panels A1 and C1) and were significantly reduced in p62-LAB treated mice (Figure 2, panel E1). Similarly, the strong labeling of the amyloid peptide in the spleen of control and LAB mice (Figure 2, panels A2 and C2) resulted decreased upon treatment (Figure 2, panel E2). As for the brain, p62-LAB exposure (Figure 2, panel E3) markedly reduced the clear Aβ(1–42) deposition observed in untreated mice (Figure 2, panel A3). A less evident but significant decrease of this amyloid peptide was also detected upon LAB exposure (Figure 2, panel C3). Interestingly, the double staining analysis clearly indicated a correlation between the increased levels of endogenous p62 and the reduction in Aβ(1–42) peptide. These data on the diminished levels of the amyloid peptide were also confirmed by additional IHC analysis performed on brain sections stained with the 6E10 antibody that reported a reduction of amyloid amounts upon p62-LAB treatment (Supplementary Figure 3). Both Aβ(1–42) and Aβ(1–40) peptides were quantified in brain homogenates by ELISA and lower levels were observed upon treatment with p62-engineered *Lactobacilli* compared to C and LAB groups (Figure 4, panels A and B, respectively). In detail, the most evident effect was detected on the Aβ(1–42) peptide (42% decrease compared to controls) whose concentration was also partially reduced by the treatment with LAB (30% decrease compared to controls). As expected, 16-weeks-old untreated mice showed higher brain amounts of both peptides compared to T0 group: 1.57- and 1.52-fold increase for Aβ(1–42) and Aβ(1–40), respectively.

### Effects of p62-LAB treatment on neuronal proteolysis

**Figure 5:**
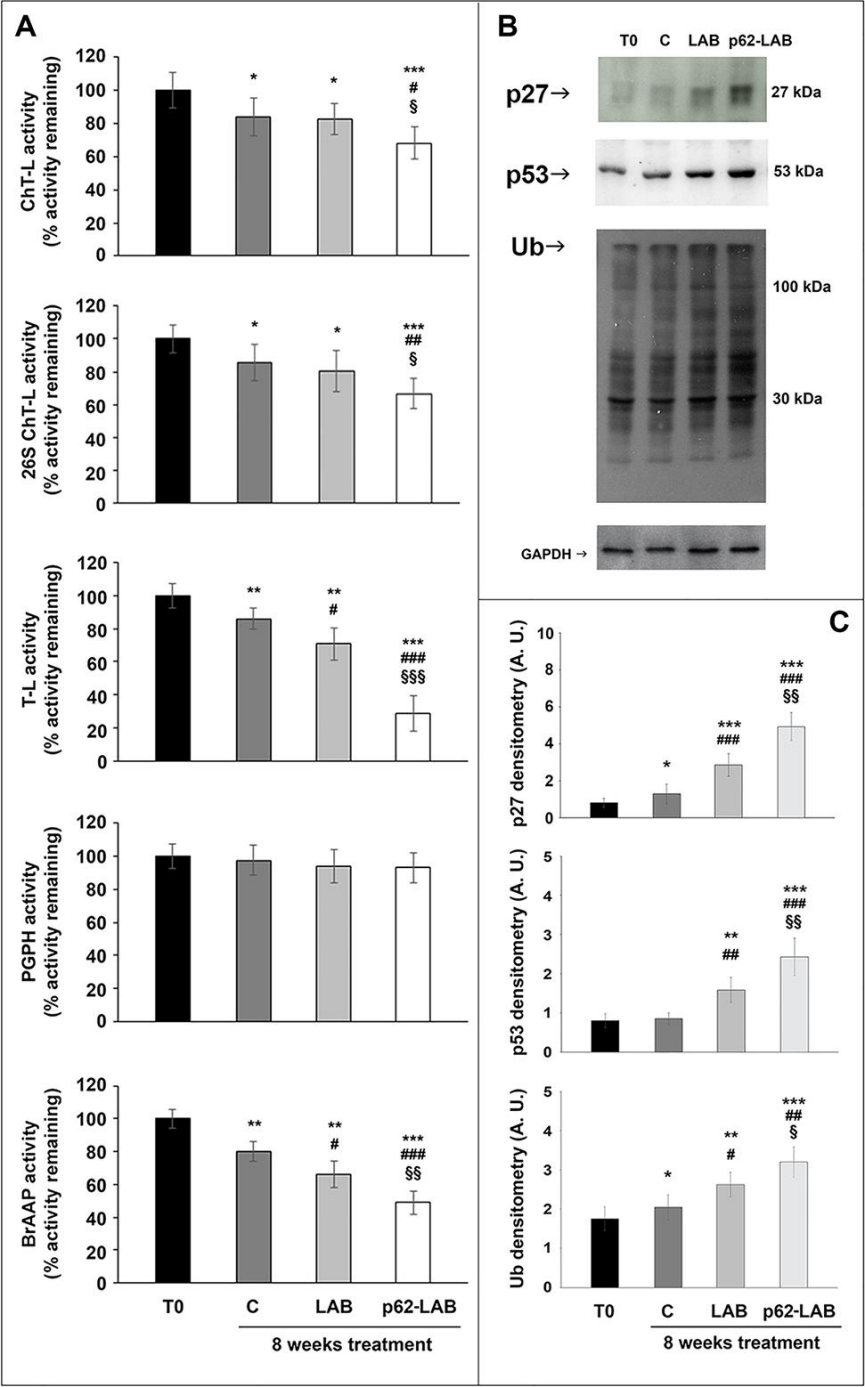
**Proteasome functionality in brain homogenates of 3xTg-AD mice.** The ChT-L, T-L, PGPH and BrAAP components of the 20S proteasome and the ChT-L activity of the 26S proteasome were measured using fluorogenic peptides as reported in the Material and Method section (panel **A**) (^*^p<0.05, ^**^p<0.01 and ^***^p<0.001 vs T0, ^#^ p<0.05, ^##^ p<0.01 and ^###^p<0.001 vs C, ^§^p<0.05, ^§§^p<0.01 and ^§§§^p<0.001 vs LAB). Immunodetection of the proteasome substrates p27, p53 and Ub-protein conjugates in brain homogenates of 3xTg-AD mice, T0, C, LAB and p62-LAB groups (panel **B**) and related densitometry (panel **C**). GAPDH was used as a control to check equal protein loading (^*^p<0.05, ^**^p<0.01 and ^***^p<0.001 vs T0, ^#^p<0.05, ^##^ p<0.01 and ^###^p<0.001 vs C, ^§^p<0.05 and ^§§^p<0.01 vs LAB).

Concerning cellular proteolysis, the p62 protein drives substrates to the UPS or autophagy for degradation [16, 21]. We explored whether the administration of p62-engineered LAB could modulate brain proteolysis, with a special emphasis on these two major proteolytic systems. As expected, an age-dependent decrease in proteasome activity was detected in brain homogenates of the control group when compared to T0 animals (Figure 5, panel A). Compared to control mice, only the T-L activity was significantly decreased in the LAB treated group, whereas an evident subunit-dependent inhibition of the complex was observed in p62-LAB treated mice, with the T-L and BrAAP activities as the most influenced by the treatment (70% and 50% inhibition, respectively). Only the PGPH subunit was not affected by the treatment (Figure 5, panel A). The inhibition of this enzymatic system in the brain of p62-LAB treated animals was also confirmed by the accumulation of the proteasomal substrates p27, p53 and ubiquitinated proteins (Figure 5, panel B).

**Figure 6:**
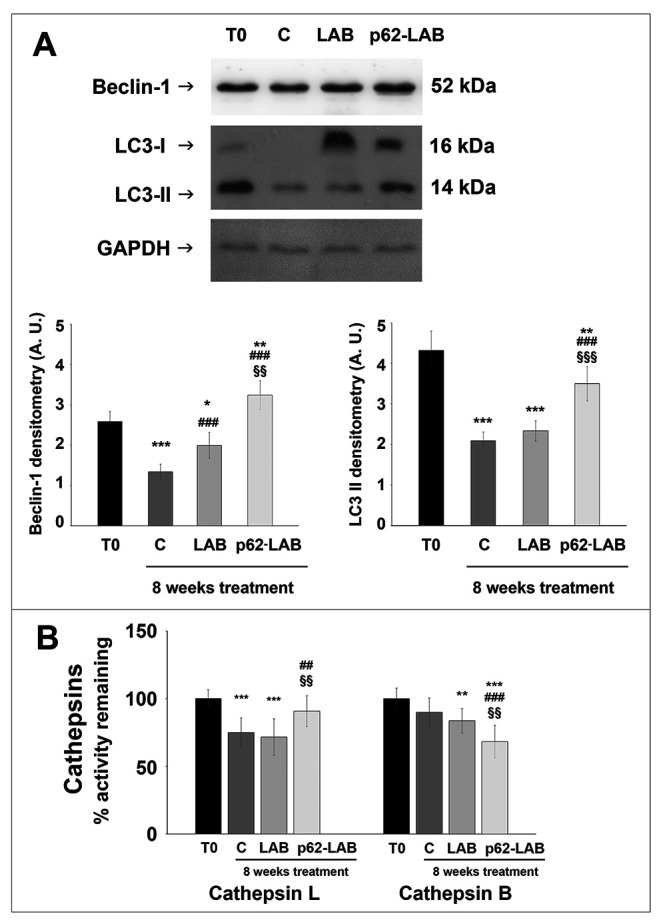
**Autophagic markers in the brain of 3xTg-AD mice.** Beclin-1 and LC3 II expression in brain homogenates of 3xTg-AD mice (panel **A**). GAPDH was used as a control to check equal protein loading (^*^p<0.05, ^**^p<0.01 and ^***^p<0.001 vs T0, ^###^p<0.001 vs C, ^§§^p<0.01 and ^§§§^p<0.001 vs LAB). Cathepsin L and B activity measured in brain homogenates of 3xTg-AD mice, T0, C, LAB and p62-LAB groups (panel **B**) (^**^p<0.01 and ^***^p<0.001 vs T0, ^##^p<0.01 and ^###^p<0.001 vs C, ^§§^p<0.01 vs LAB).

To evaluate the effects on autophagy, we immunodetected the levels of the autophagy-related proteins beclin-1, involved in the recruitment of membranes to form autophagosomes, and LC3 II, the lipidated form of LC3, localized in autophagosomal membranes. The expression of both proteins was significantly up-regulated by p62-LAB treatment and an increased conversion of LC3-I into LC3-II was detected (Figure 6, panel A). Then, to estimate lysosomal function, we measured the activity of two major lysosomal hydrolases, cathepsin B and cathepsin L (Figure 6, panel B) [22]. Cathepsin B (Cat B) was previously associated with amyloid plaques and related memory loss and its inhibition was suggested to reduce Aβ levels [23, 24]. Interestingly, a 25% inhibition of Cat B activity was observed in p62-LAB treated mice compared to 16-weeks-old untreated mice (group C). Conversely, cathepsin L (Cat L), whose activity was reduced in control and LAB-treated animals compared to T0 group (29% and 26% inhibition, respectively), was significantly restored by p62-LAB exposure (21% activation respect to C) (Figure 6, panel B). This finding is of extreme importance considering Cat L role in the production of numerous peptide neurotransmitters and its α-secretase activity that is known to suppress amyloid peptides levels [25].

### Effects of p62-LAB treatment on macromolecules oxidation

**Figure 7:**
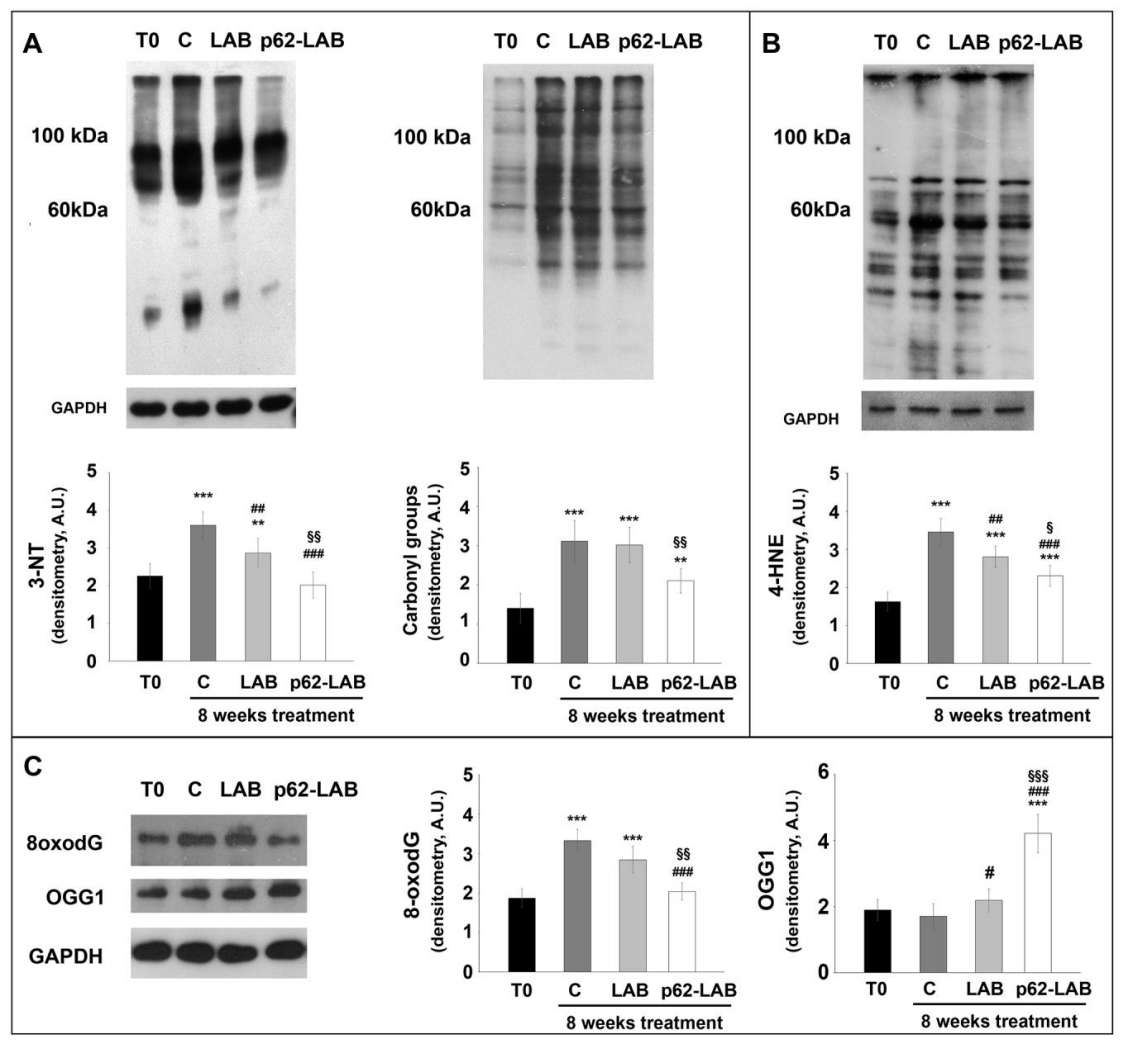
**Treatment effects on proteins, lipids and DNA oxidation.** Determination of carbonyls and 3-NT (panel **A**), 4-HNE (panel **B**), 8-oxodG and OGG1 (panel **C**) in brain homogenates of 3xTg-AD mice (T0, C, LAB and p62-LAB groups). GAPDH was used as a control to check equal protein loading (*p<0.05, **p<0.01 and ***p<0.001 vs T0, ^##^p<0.01 and ^###^p<0.001 vs C, ^§^p<0,05, ^§§^p<0.01 and ^§§§^p<0.001 vs LAB).

Extensive oxidative damage to cellular macromolecules characterizes the AD brain and p62 protein plays a protective role in oxidative conditions [26, 27]. Homogenized brain tissues from T0, control, LAB and p62-LAB treated mice were analyzed for the detection of oxidative markers. Protein oxidation was evaluated measuring carbonyls and 3-nitrotyrosine (3-NT) levels and results are reported in figure 7, panel A. The treatment with the p62-transformed bacteria was successful in reducing the amount of both oxidation products with a 1.78- and a 1.37-fold decrease of 3-NT and carbonyls compared to control, respectively. Similar results were obtained for 4-hydroxy-2-nonenal (4-HNE), a product of lipid peroxidation, whose levels where strongly reduced upon mice treatment with engineered LAB (-65% compared to control, Figure 7, panel B). DNA oxidation was determined detecting 8-oxo-7,8-dihydro-2′-deoxyguanosine (8-oxodG), the most prevalent form among oxidative base modifications, and the expression of 8-oxoguanine DNA glycosylase-1 (OGG1) responsible for the removal of oxidized guanine base lesions generated by free radicals [28]. Interestingly, lower levels of 8-oxodG were detected in p62-LAB treated mice (1.6-fold reduction compared to control animals) that also showed a parallel increased expression of the DNA repair enzyme (2.4-fold increase) (Figure 7, panel C).

### Effects of p62-LAB treatment on inflammation

**Figure 8:**
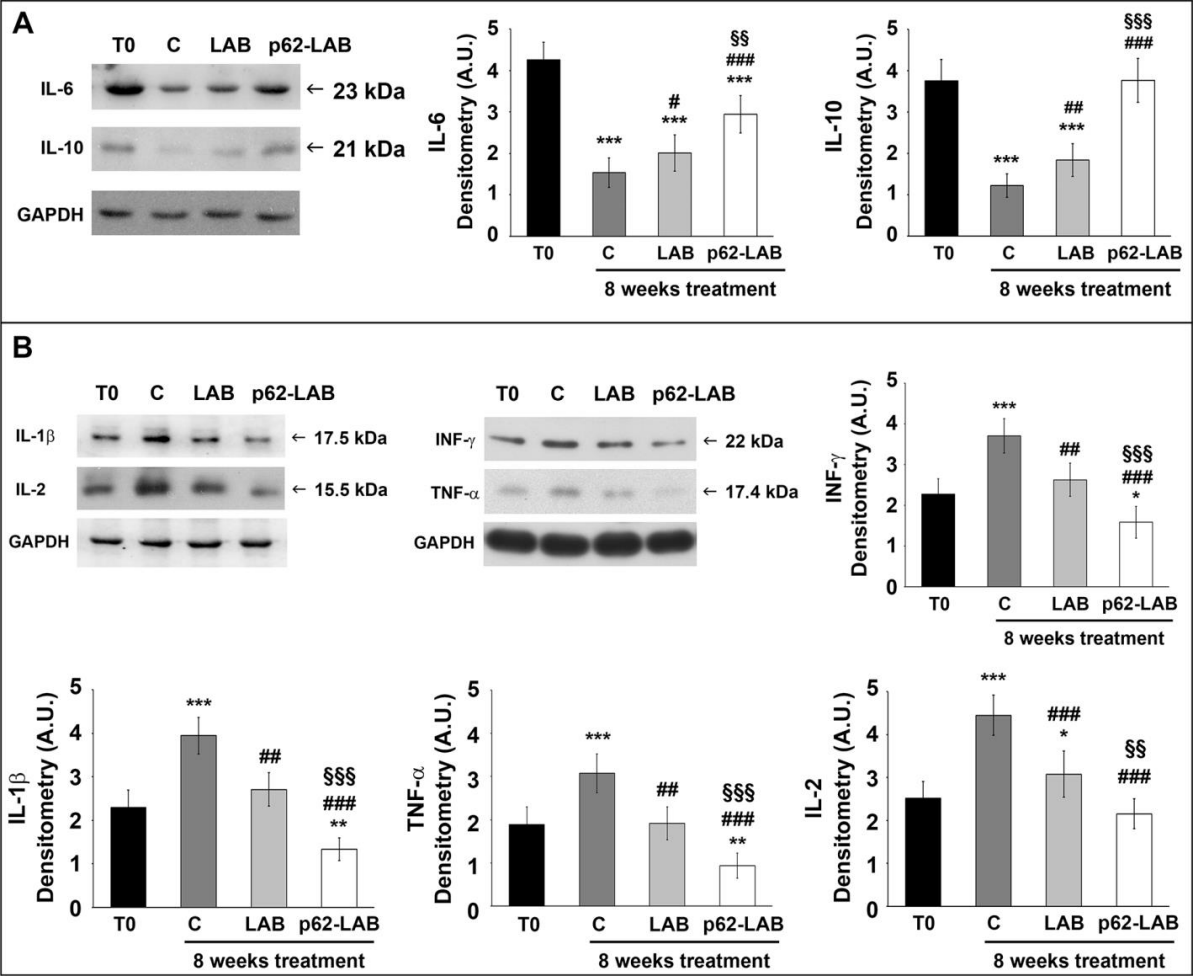
**Treatment effects on brain anti- and pro-inflammatory cytokines.** Detection of anti-inflammatory (panel **A**) and pro-inflammatory (panel **B**) cytokines by Western blot analysis in brain homogenates of 3xTg-AD mice (T0, C, LAB and p62-LAB groups). GAPDH was used as a control to check equal protein loading (*p<0.05 and ***p<0.001 vs T0, ^#^ p<0.05, ^##^ p<0.01 and ^###^p<0.001 vs C, ^§§^p<0.01 and ^§§§^p<0.001 vs LAB).

A panel of pro- and anti-inflammatory cytokines was monitored performing immunoassays to evaluate the modulatory effect of the treatment on brain inflammation (Figure 8). Our data definitely demonstrated that transformed LAB were able to significantly reduce the inflammatory status in AD mouse brain by stimulating the expression of anti-inflammatory molecules (Figure 8, panel A) and the simultaneous decrease of pro-inflammatory cytokines (Figure 8, panel B). In detail, the most consistent up-regulation for the anti-inflammatory cytokines was measured for IL-10 (3-fold increase compared to control). Conversely, all the pro-inflammatory molecules, that were heavily up-regulated with age (T0 vs C), resulted significantly reduced upon p62-LAB treatment (2.35-, 2.95-, 3.25- and 2.0-fold decrease for INF-γ, IL-1β, TNF-α and IL-2, respectively, compared to control). A weaker but still significant effect was also observed in mice administered with LAB.

### Effects of p62-LAB treatment on components of the gut-brain axis

Considering the ability of probiotics to exert neuroprotective effects through the modulation of components of the gut-brain axis [5], we measured the plasma concentration of gut peptide hormones and the microbiota composition in the four experimental groups. An age-dependent decrease of glucose-dependent insulinotropic polypeptide (GIP), glucagon like peptide-1 (GLP-1) and ghrelin concentration was detected in untreated AD mice (C) but no statistically significant changes were observed upon LAB or p62-LAB treatment (Supplementary Figure 4).

**Figure 9:**
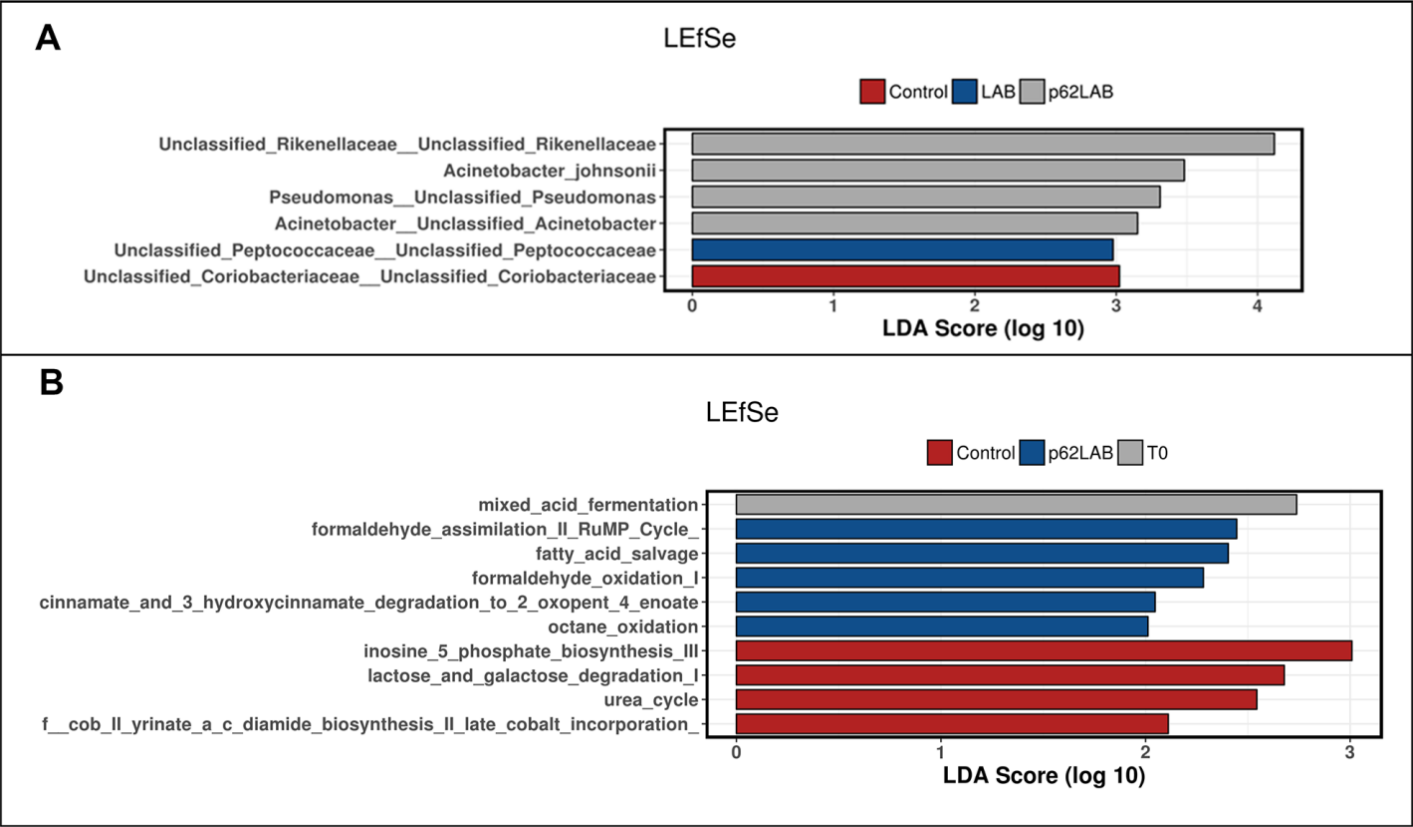
**Linear discriminant analysis effect size (LefSe) of bacterial taxa and KEEG pathways.** LefSe of bacterial taxa and their association with C, LAB and p62-LAB groups. Only LefSe values >2 are shown (panel **A**). LEfSe of the differentially abundant KEGG pathways in T0, C and p62-LAB groups. Only LefSe values >2 are shown (panel **B**).

Microbiota composition was screened to check for changes in richness, the number of species present in a sample, and evenness, the related differences in abundance of species. Interestingly, the analysis of bacterial communities showed no significant difference in alpha diversity indices (Observed species, Chao1, and Shannon index), revealing no change in species richness and evenness (data not shown). Principal coordinate analysis (PCoA) based on unweighted Unifrac distance metric was used to assess beta-diversity, a parameter that compares bacterial communities among samples. The results obtained did not reveal significant clustering between groups (ANOSIM_Unweighted_: *R* = 0.003, *p* = 0.475, data not shown). Linear discriminate analysis effects size (LEfSe) detected few differentially abundant bacterial taxa on the phylogenetic levels (Figure 9). At the species level, the most evident change induced by the p62-LAB treatment was the increased abundance of unclassified species belonging to *Rikenellaceae* family (LDA score >4.0), whereas an increase in unclassified species belonging to *Peptococcaceae* family was associated to LAB administration (LDA score >2.9) (Figure 9, panel A). Univariate statistics revealed no significant differences between groups at phylum and class levels and only few differences at the other taxonomic levels including a decrease in *Flexispira* and in *Erysipelotrichaceae* in the p62-LAB group (C vs p62-LAB, p<0.0093 and p<0.0409, respectively) and an increase in *f_Ruminococcaceae* in both LAB and p62-LAB group (C vs LAB 0.0455, C vs p62-LAB 0.0252). PICRUSt analysis to determine the predicted metabolic functions of the microbial communities detected changes in five pathways, with a LDA score between 2.0 and 2.4, in the gut microbiome of p62-LAB group (Figure 9, panel B). In addition, ten metabolic pathways differed with a p<0.05 between C and p62-LAB groups (Supplementary Table 1).

## DISCUSSION

AD is an age-associated disease characterized by numerous pathological hallmarks that slowly destroy memory and learning ability. Irrespective of considerable efforts made to develop new effective therapeutic agents, the drugs that are currently approved for the treatment of this disorder only provide symptomatic relief but cannot prevent the onset or block the progression of the pathology. Considering the multifactorial nature of AD, the design of novel drugs with a broad spectrum of activity could be a successful strategy for preventing its development. Recently, the use of recombinant bacteria, mainly *Lactobacilli*, has received great interest for their ability to act as delivery vectors of therapeutic molecules to mucosal surfaces [29]. These recombinant cells were tested in mouse models of colitis to reduce inflammation and reestablish intestinal homeostasis and of type 1 diabetes to restore antigen-specific tolerance [29].

In the view of these promising results, this work investigated the neuroprotective effects of a *Lactobacillus lactis* strain engineered with the pExu plasmid encoding the human p62 protein (pExu:p62 vector). This protein is widely recognized for its multiple roles in the cell and also emerged as a key factor in AD [30]. In addition, previous data demonstrated that the administration of a p62-encoding plasmid exerted a powerful anti-osteoporotic, anti-inflammatory, and retinoprotective activity in animal models [31, 32].

Herein, we observed a marked increase in endogenous p62 levels in different tissues of p62-LAB treated mice. Interestingly, a weak staining of the human p62 protein was also detected, confirming previous findings on the ability of these bacteria to behave like vectors for local delivery of molecules to the gastrointestinal tract [10, 33]. The analysis of the distribution of the p62 protein in p62-LAB treated animals performed by immunohistochemistry indicates the classical way of delivery of the protein from the gut mucosa to the brain, by lymphatic vessels and some neural terminations. In fact, a progressive decreased expression of the exogenous human-p62 peptide together with an increase of the endogenous mouse p62 protein were observed in the mesenteric lymph-nodes, in the spleen and in the brain. This dissemination route was also described in other disorders in which the infectious agents, such as viruses or bacteria, can gain access to the brain by using peripheral nerves (neural neuroinvasion) or the lymphatic/hematic way [34]. In our case, as shown by the results of IHC, the p62 protein follows both lymphatic and axonal pathways to finally reach the central nervous system (CNS). Comparatively, results from experimental sheep oral infections with the prion disease agent PrP^sc^ showed that it reaches the brain through the early involvement of the enteric and abdominal ganglia. Data on the diffusion of the scrapie infectious agent described a three steps-based accumulation process upon oral ingestion of the prion protein: a first phase, with the accumulation in gut-associated lymphoid tissue (GALT), a second step involving the lymphatic dissemination of the protein to non-GALT lymphatic tissues and a final neuro-invasion phase until the CNS [35–37].

Considering the numerous evidences associating decreased levels of p62 with AD pathology, it is reasonable to hypothesize that a reestablishment of its concentration could counteract AD signs [20, 30]. This assumption was confirmed by the NOR test that demonstrated a significant improvement in the recognition memory of treated mice. These encouraging outcomes positively correlated with lower brain deposition of APP and Aβ(1–40) and Aβ(1–42) peptides, the major agents responsible for plaques formation. Exploring neuronal proteolysis, p62-LAB treated animals showed proteasome inhibition and the simultaneous activation of autophagy, with increased LC3 and beclin-1 levels and selective modulation of cathepsin B and L activity. It is widely known that the two proteolytic pathways are closely related to each other and p62 is an important modulator of their cross-talk, and its presence contributes to the maintenance of protein homeostasis [5, 16, 38–41]. Previous evidences described compensatory mechanisms between the two systems, with proteasome inhibition favoring autophagy stimulation [38, 40]. Increased autophagy and overexpression of beclin-1 and LC3 were previously demonstrated to exert neuroprotective effects favoring amyloid-beta protein clearance in AD mouse models [20, 42–46]. Autophagy is a highly dynamic multi-step process that can be modulated at different steps [47]. Thus, the expression of autophagy associated proteins is not always easy to interpret and several parameters must be considered. Our findings show that increasing p62 concentration upregulates autophagy-related proteins LC3II and beclin-1 and modulates cathepsins activity to finally promote autophagic removal of amyloid peptides. In particular, our results are in line with the findings of Caccamo et al., reporting that increasing brain expression of p62 favors the autophagy-mediated Aβ degradation [20]. However, differently from their gene therapy approach with direct injection of DNA into ventricles of newborn pups, we propose a non-toxic and non-invasive oral therapy.

Oxidative damage and inflammation are important components of AD brain pathology. Specifically, oxidation is observed in membranes, proteins and nucleic acids and associates with their loss of function whereas inflammatory alterations are accompanied by increased levels of pro-inflammatory cytokines [48–50]. Here, we demonstrated that p62-LAB oral administration induces a clear reduction of oxidative and inflammatory markers, which is consistent with the previously identified anti-inflammatory and anti-oxidant properties of p62 [51–54]. In addition, since Aβ is a direct source of oxidative stress and that its deposition is linked to microglia activation and sustained pro-inflammatory signaling, reduction of its levels further contributes to counteract the two detrimental processes [48, 50, 55, 56]. A less evident but significant effect was observed upon treatment with control lactic acid bacteria that emerged as particularly successful in reducing inflammatory markers, thus confirming the health benefits associated to probiotic consumption [5, 57–59]. However, their use as delivery vectors for the p62-pExu plasmid undoubtedly strengthens such effects and provides additional support to the therapeutic action of the proposed strategy in AD.

The analysis of microbiota composition revealed no side effects on gut microbiota nor major changes in composition in treated mice, with only a few taxa being shifted. Interestingly, in accordance with its anti-inflammatory properties, p62-LAB treatment decreased the relative abundance of the family *Erysipelotrichaceae*, previously demonstrated to be increased with age in AD mice and with a positive correlation with a pro-inflammatory gut micro-environment. In addition, an increase in the family *Ruminococcacea*, one of the major butyrate producing families and, remarkably, inversely correlated with intestinal permeability in AD, was observed [60–63]. The upregulated KEGG pathways were mainly related to the degradation of aromatic compounds that can be used as a source of carbon and energy, upon their conversion to substrates of the tricarboxylic acid cycle. Moreover, the absence of changes in gut hormones and the minor modifications in microbiota composition and related metabolic pathways suggest that the observed effects do not depend upon the modulation of gut-brain axis components.

Conclusively, our data demonstrate the therapeutic potential of p62-engineered *Lactobacilli* in an AD mouse model and propose a novel multi-target approach for AD treatment that takes advantage of the multifunctional activity of the p62 protein and of the beneficial properties and safety of these food grade bacteria. Nevertheless, additional pre-clinical studies are required to evaluate the long-term effects of the treatment and the persistence of the obtained results after the treatment is discontinued. Furthermore, once addressed the safety concerns on the use of engineered strains by humans and validated the strategies to optimize biocontainment and the translational potential of these modified microbes [64], clinical trials will be important steps to confirm the effective benefit of this approach for human AD patients. In fact, despite 3xTg-AD mice are a widely accepted model for human AD and despite mouse and human share numerous similarities in physiology and anatomical structures, we should take into account that minor differences in the gastrointestinal tract (including microbiota composition), due to genetic background and environmental factors, could influence the final outcome of the study.

## MATERIALS AND METHODS

### Engineered bacterial cells growth and lyophilization

The *Lactobacillus lactis* subsp. *cremoris* MG1363 strain, both control (LAB) and p62-pExu transformed cells (p62-LAB), were obtained from the laboratory of Prof. Azevedo at the Federal University of Minas Gerias (Belo Horizonte, Brazil) [33]. These cells were maintained as stock cultures in glycerol at -80°C until use. Transformation of *L. lactis*, pExu plasmid construction and *in vitro* and *in vivo* functionality evaluation were described by Mancha-Agresti et al. [33]. The p62 ORF fragment was inserted into pExu MCS using the restriction enzymes, resulting in pExu:p62. The pExu:p62 plasmid was then established by transformation in *L. lactis* MG1363 strain [33]. Two populations of *L. lactis* subsp. *cremoris* MG1363 were grown: LAB added with the pExu:empty plasmid (indicated as LAB), LAB added with the pExu vector encoding for human p62 (pExu:p62, indicated as p62-LAB).

*L. lactis* subsp. *cremoris* MG1363 was grown in M17 medium (Sigma-Aldrich) containing 0.5% glucose (Sigma-Aldrich) and 125 μg/mL erythromycin (Sigma-Aldrich) at 30 °C without agitation. For bacteria lyophilization, culture medium containing 5×10^8^ CFU/mL (corresponding to 1 O.D.) was centrifuged and the supernatant discarded. Pellet was suspended in pasteurized skimmed milk and aliquoted in 2 mL tubes each containing the daily dose of bacteria for 8 mice (8×10^9^ cells). Tubes were frozen and then dried for 24 h. Cell viability and number were checked upon lyophilization. Lyophilized bacteria were stored at 4° C.

### Animals and treatment

Triple transgenic mice 3xTg-AD were purchased from Jackson Laboratory (Bar Harbor, Maine, USA). This is a widely used animal model for AD due to its three distinguished mutations corresponding to familial AD (APP Swedish, tau P301L, and PSEN1 M146V). Translation of the overexpressed transgenes are confined to the central nervous system, including the hippocampus and cerebral cortex. Aβ deposition is continuous, with first signs surfacing within 3 to 4 months of age in the frontal cortex and becoming more extensive by 12 months [65, 66]. 8-weeks-old male mice were divided into four groups as follows: untreated 8-weeks-old mice (T0, n=8); mice treated with lyophilized milk (C, n=8), mice treated with control lyophilized LAB (pExu:empty, n=8) and mice treated with lyophilized p62-LAB (LAB(pExu:p62), n=8). Bacteria were dissolved daily in mice drinking water and given to the animals. Mice received 10^9^ bacteria every day. After a two-month treatment, mice were sacrificed. According to the guidelines of the European Communities Council (86/609/ECC) for the care and use of laboratory animals, mice were maintained in a temperature-controlled room (21±5 °C) under an inverted 12 h light/dark cycle (lights on at 8:00 pm) and provided with rodent standard food (Mucedula, Italy) and water *ad libitum*. All appropriate measures were taken to minimize pain and discomfort in these experimental animals. General observations regarding possible changes in skin and fur, mucous membranes, diarrhea, sleep, movements and posture were performed. Additionally, local injuries and mortality (if any) were recorded throughout the experimental period. The body weight was measured at the beginning of the treatment and then once a week to ensure adequate food intake.

### Novel object recognition

Behavioral tests were performed during the animals’ dark phase starting from 8:00 to 15:00. Animals were handled for 3 days before testing in order to accustom them to the experimenter. The investigators were blinded to the groups’ allocation during the tests. The novel-object recognition test (NOR, based on the spontaneous tendency of rodents to spend more time exploring a novel object than a familiar one) was used to evaluate recognition memory. The first step is the habituation, during which the animal is allowed to explore the empty arena for 5 min and then returns to the home cage. Following a training period dedicated to the exploration of two identical objects, the animal is removed from the arena for a delay period of 3 h, and it is then placed back within the arena, where one of the two identical objects is replaced by a new, dissimilar novel object (test phase). The time the rodent spends exploring each object in 10 min provides a measurement of the extent of memory integrity and attention. Results were expressed as discrimination scores (the ratio between the time spent with novel object and total time spent with both objects). A lower score indicates memory impairment in this task. Objects were different in shape, color and texture and maintained throughout the study to obtain reproducible data. Preliminary experiments were done to select novel and familiar object pairs, so that each object in the pairs elicited the same amount of spontaneous investigation.

### Mice sacrifice and tissues preparation

After the treatment, mice were sacrificed, and feces frozen for microbiota determinations. Blood and organs were collected, brains weighed, dissected and photographed, and immediately frozen or fixed in 10% buffered formalin for morphological analyses and immunohistochemistry. A portion of the brains was homogenized (1:5 weight/volume of buffer) in 50 mM Tris buffer, 150 mM KCl, 2 mM EDTA, pH 7.5. Homogenates were immediately centrifuged at 13000×g for 20 min at 4 °C and supernatants collected for enzymatic assays and western blotting. An aliquot of the supernatants was supplemented with protease inhibitors (Pefabloc and TPCK) for ELISA determinations. The Bradford method was used to measure the protein concentration in homogenates, using bovine serum albumin (BSA) as a standard [67].

### ELISA assay for Aβ levels

Brain homogenates (supernatant fraction) supplemented with protease inhibitors (Pefabloc and TPCK), were used to measure Aβ(1–40) and Aβ(1–42) levels using enzyme-linked immunosorbent assay NOVEX^®^ ELISA kits (Thermo Fisher Scientific Inc., Waltham, MA USA). Based on preliminary tests, samples were diluted at 1:5 with diluent buffer provided with the kit. Plates were read at 450 nm on a visible plate reader (Biotrak, Amersham). Assays were performed according to the manufacturer’s directions.

### Immunohistochemistry

For immunohistochemistry, brains, intestines, mesenteric lymph-nodes, and spleens were collected, formalin fixed, paraffin embedded and 3 μm sections were prepared. Murine and human p62 expression levels were measured using two distinct anti-p62/SQSTM1 antibodies from Cell Signaling Technology (Leiden, The Netherlands, dilution 1:100), the anti-rodent p62/SQSTM1 antibody, produced in rabbit against synthetic peptides corresponding to residues surrounding Gly-300 of mouse p62 protein, and the anti-human p62/SQSTM1 protein antibody, produced against synthetic peptides corresponding to residues surrounding Pro-220 of human p62 protein. Aβ(1-42) deposition was evidenced and scored using the anti-Aβ(1-42) antibody from Merck Millipore (Merck KGaA, Darmstadt, Germany). The anti-Aβ (6E10) antibody was purchased from BioLegend (BioLegend Way San Diego, CA, USA). For double-labeling immunohistochemistry, slides were first incubated with the anti-Aβ(1-42) antibody and with the anti-murine p62/SQSTM1 antibody and then with the biotin-labeled goat anti-rabbit secondary antibody (1:200, Jackson ImmunoResearch, West Grove, PA). The binding of the antibody was detected with the Elite kit (Vector Laboratories) and the immunoreaction was developed using two different chromogens: violet (VIP, Vector, Burlingame UK) for mouse and human p62 protein stain and brown (DAB, Vector) for anti-Aβ(1-42) antibody. Tissues were counterstained with Mayer’s hematoxylin. For negative immunohistochemical controls, the primary antibodies were omitted. Lower-power digitized images were acquired with a BX-60 microscope (Olympus, Melville, NY) equipped with a DEI-470 digital camera (Optronics, Goleta, CA). The immunoreactivity in the tested areas was quantified at the indicated magnification and analyzed using ImageJ/Fiji 1.52p software (NIH, USA) [68] defining a region of interest (ROI) for the colors to be measured. The absence of primary antibody did not result in immunoreactivity. The pathologist was blinded to the group allocation.

### Western blot analysis

Brain homogenates (20 μg of proteins) were loaded on 12% SDS-PAGE and electroblotted onto polyvinylidene fluoride (PVDF) membranes. Membranes were activated with methanol and blocked with 5% BSA in freshly prepared TTBS (Tween 20 plus Tris-HCl and NaCl, pH 7.5). Antibodies were diluted in 2% BSA in TTBS. Proteins were detected with the enhanced chemiluminescence (ECL) system (Amersham Pharmacia Biotech, Milano, Italy). The anti-rodent p62/SQSTM1 antibody and the anti-human p62/SQSTM1 protein antibody were purchased from Cell Signaling Technology (Leiden, The Netherlands). The anti-Aβ(1-42) antibody was from Merck Millipore (Merck KGaA, Darmstadt, Germany). Recombinant human p62/SQSTM1 protein was obtained from Abcam plc (Cambridge, UK). The anti-LC3 antibody was from Thermo Fisher Scientific (MA, USA). The intracellular levels of beclin-1, p27, p53, ubiquitin-conjugates, 3-nitrotyrosine (3-NT), 4-hydroxy-2-nonenal (4-HNE), 8-oxo-7,8-dihydro-2′-deoxyguanosine (8-oxodG) and 8-oxoguanine DNA glycosylase-1 (OGG1) were determined using primary antibodies from Santa Cruz Biotechnology, in accordance to their related protocol/information sheet. Primary antibodies used to detect pro- and anti-inflammatory cytokines were from Abcam plc (Cambridge, UK). Membranes for western blotting analyses were purchased from Millipore (Milano, Italy). Molecular weight markers (6.5 to 205 kDa) were included in each gel. Glyceraldehydes-3-phosphate dehydrogenase (GAPDH) was used as a control to check equal protein loading [69]. Membranes were stripped using a stripping buffer containing 200 mM glycine, 0.1% SDS and 1% Tween 20. Immunoblot images were quantified using ImageJ 1.52p software (NIH, USA).

### Oxyblot analysis

Carbonyl groups in proteins were measured with the Oxyblot kit (Appligene-Oncor, Strasbourg, France). Briefly, brain homogenates (15 μg of total proteins) were incubated at room temperature with 2,4-dinitrophenylhydrazine (DNPH) in order to form 2,4-dinitrophenylhydrazone (DNP-hydrazone), according to the manufacturer data sheet. Then, the obtained products were separated by SDS-PAGE and electroblotted onto PVDF membranes. The detection step and data analysis were performed as described in the Western Blot Analysis section [70].

### Proteasome activity assays

Proteasome peptidase activities in brain homogenates (supernatant fraction) were determined using the synthetic fluorogenic peptides Suc-Leu-Leu-Val-Tyr-AMC for ChT-L activity, Z-Leu-Ser-Thr-Arg-AMC for T-L activity, Z-Leu-Leu-Glu-AMC for PGPH activity, and Z-Gly-Pro-Ala-Phe-Gly-pAB for BrAAP activity [71]. The mixture contained the brain homogenate (15 μg total proteins), the appropriate substrate (5 μM final concentration) and 50 mM Tris–HCl pH 8.0, up to a final volume of 100 μL, and was incubated at 37 °C for 60 min. The 26S proteasome ChT-L activity was tested including 10 mM MgCl_2_, 1 mM dithiothreitol, and 2 mM ATP in the reaction mix. The fluorescence of the hydrolyzed 7-amino-4-methyl-coumarin (AMC) and 4-aminobenzoic acid (pAB) (AMC, λ_exc_ = 365 nm, λ_em_ = 449 nm; pAB, λ_exc_ = 304 nm, λ_em_ = 664 nm) was detected on a SpectraMax Gemini XPS microplate reader.

### Cathepsin B and L activities

Cathepsin B and L proteolytic activities were measured using the fluorogenic peptides Z-Arg-Arg-AMC and Z-Phe-Arg-AFC, respectively, at a final concentration of 5 μM [40]. The mixture for cathepsin B, containing 7 μg of brain homogenate, was pre-incubated in 100 mM phosphate buffer pH 6.0, 1 mM EDTA and 2 mM dithiothreitol for 5 min at 30 °C. Upon the addition of the substrate, the mixture was incubated for 15 min at 30 °C. The mixture for cathepsin L, containing 7 μg of proteins, was incubated in 100 mM sodium acetate buffer pH 5.5, 1 mM EDTA and 2 mM dithiothreitol for 5 min at 30 °C and, upon the addition of the substrate, the mixture was incubated for 15 min at 30 °C. The fluorescent signal released by the hydrolyzed 7-amino-4-methyl-coumarin (AMC, λ_exc_ = 365 nm, λ_em_ = 449 nm) and 7-amino-4-trifluoromethylcoumarin (AFC, λ_exc_ = 397 nm, λ_em_ = 500 nm) was detected on a SpectraMax Gemini XPS microplate reader.

### ELISA assay for ghrelin, leptin and GIP, GLP-1

Plasma hormone concentrations were measured through ELISA using plasma treated with protease inhibitors (Pefabloc and TPCK). The Rat/mouse Ghrelin Active ELISA kit (Invitrogen) is a sandwich ELISA based on the capture of ghrelin molecules (active form) in the plasma by anti-ghrelin IgG and the immobilization of the resulting complex to the wells of a microtiter plate coated by a pre-titered amount of anchor antibodies. Leptin and glucose-dependent insulinotropic polypeptide (GIP) (Merck KGaA, Darmstadt, Germany) were determined using sandwich ELISA kit based on anti-leptin and anti-GIP monoclonal antibodies, respectively. Similarly, the quantitative determination of mouse glucagon like peptide-1 (GLP-1) was performed using a sandwich ELISA kit (CUSABIO, Houston, Texas). Plates were read on a visible plate reader (Biotrak, Amersham). Assays were performed following manufacturer’s indications.

### 16S rRNA gene sequencing

Bacterial DNA extraction from fecal samples was performed using a commercially available kit according to the manufacturer’s instructions and as described elsewhere [72]. Bacterial tag-encoded FLX-titanium amplicon pyrosequencing (bTEFAP) based on the V1–V3 region (*E. coli* position 27–519) of the 16S rRNA gene was performed on fecal samples from mice (groups T0, C, LAB and p62-LAB) as described previously [73], with forward primer 515F (GTGCCAGCMGCCGCGGTAA) and reverse primer 806RB (GGACTACNVGGGTWTCTAAT). Sequence data were uploaded into the NCBI GenBank database under submission number SRP225114. Raw sequence data were screened, trimmed, deionized, filtered, and chimera depleted using a QIIME2 pipeline [74]. Sequence were demultiplexed and the Operational taxonomic units (OTUs) table was created using DADA2 [75]. OTUs were defined as sequences with at least 97% similarity using Greengenes v.13.8 database [76]. The OTU table was rarefied at 23,365 sequences/ sample for even depth of analysis. Phylogeny-based UniFrac distance metric analysis was used as a measure of beta (β)-diversity to investigate differences in microbial communities. For this, the analysis of similarity (ANOSIM) function in Primer6 was used on the UniFrac distance matrixes, both weighted and unweighted. Alpha (α) diversity was assessed as a measure of species richness and evenness in all samples. The Chao1, Shannon index and Observed Species data and plots were generated. Univariate statistics were performed on alpha-diversity data and on OTU tables with Kruskal-Wallis, using Graphpad Prism 7. P-values were adjusted for multiple comparisons by the Benjamin and Hochberg FDR. Statistical significance was set at p<0.05. Post hoc Dunn's multiple comparison test was used to determine the group differences in bacterial taxa.

PICRUSt (Phylogenetic Investigation of Communities by Reconstruction of Unobserved States) was used to predict functional gene content based on 16S rRNA gene data present in the Greengenes database and the KEGG database [77] using QIIME2. Linear discriminant analysis effect size (LEfSe) was used to elucidate bacterial taxa and genes that were associated with each treatment. LEfSe was calculated using Calypso, a web-based software package that allows mining and visualizing of microbiome-host interactions [78].

### Statistical analyses

Data are expressed as mean values ± S.D. Statistical analysis was performed with one way ANOVA, followed by the Bonferroni post hoc test using Sigma-stat 3.1 software (SPSS, Chicago, IL, USA) and p<0.05 was considered statistically significant.

## Supplementary Material

Supplementary Materials and Methods

Supplementary Figures

Supplementary Table 1

## References

[1] Hardy JA, Higgins GA. Alzheimer’s disease: the amyloid cascade hypothesis. Science. 1992; 256:184–85. 10.1126/science.1566067 1566067

[2] Newell KL, Hyman BT, Growdon JH, Hedley-Whyte ET. Application of the National Institute on Aging (NIA)-reagan institute criteria for the neuropathological diagnosis of Alzheimer disease. J Neuropathol Exp Neurol. 1999; 58:1147–55. 10.1097/00005072-199911000-00004 10560657

[3] Ballatore C, Lee VM, Trojanowski JQ. Tau-mediated neurodegeneration in Alzheimer’s disease and related disorders. Nat Rev Neurosci. 2007; 8:663–72. 10.1038/nrn2194 17684513

[4] Kumar A, Singh A, Ekavali. A review on Alzheimer’s disease pathophysiology and its management: an update. Pharmacol Rep. 2015; 67:195–203. 10.1016/j.pharep.2014.09.004 25712639

[5] Bonfili L, Cecarini V, Berardi S, Scarpona S, Suchodolski JS, Nasuti C, Fiorini D, Boarelli MC, Rossi G, Eleuteri AM. Microbiota modulation counteracts Alzheimer’s disease progression influencing neuronal proteolysis and gut hormones plasma levels. Sci Rep. 2017; 7:2426. 10.1038/s41598-017-02587-2 28546539 PMC5445077

[6] Bonfili L, Cecarini V, Cuccioloni M, Angeletti M, Berardi S, Scarpona S, Rossi G, Eleuteri AM. SLAB51 probiotic formulation activates SIRT1 pathway promoting antioxidant and neuroprotective effects in an AD mouse model. Mol Neurobiol. 2018; 55:7987–8000. 10.1007/s12035-018-0973-4 29492848 PMC6132798

[7] Deng B, Wu J, Li X, Men X, Xu Z. Probiotics and probiotic metabolic product improved intestinal function and ameliorated LPS-induced injury in rats. Curr Microbiol. 2017; 74:1306–15. 10.1007/s00284-017-1318-7 28761979

[8] Kazemi A, Soltani S, Ghorabi S, Keshtkar A, Daneshzad E, Nasri F, Mazloomi SM. Effect of probiotic and synbiotic supplementation on inflammatory markers in health and disease status: a systematic review and meta-analysis of clinical trials. Clin Nutr. 2020; 39:789–819. 10.1016/j.clnu.2019.04.004 31060892

[9] Mannam P, Jones KF, Geller BL. Mucosal vaccine made from live, recombinant lactococcus lactis protects mice against pharyngeal infection with streptococcus pyogenes. Infect Immun. 2004; 72:3444–50. 10.1128/IAI.72.6.3444-3450.2004 15155651 PMC415684

[10] Chatel JM, Pothelune L, Ah-Leung S, Corthier G, Wal JM, Langella P. In vivo transfer of plasmid from food-grade transiting lactococci to murine epithelial cells. Gene Ther. 2008; 15:1184–90. 10.1038/gt.2008.59 18418419

[11] Bermúdez-Humarán LG, Langella P, Cortes-Perez NG, Gruss A, Tamez-Guerra RS, Oliveira SC, Saucedo-Cardenas O, Montes de Oca-Luna R, Le Loir Y. Intranasal immunization with recombinant lactococcus lactis secreting murine interleukin-12 enhances antigen-specific Th1 cytokine production. Infect Immun. 2003; 71:1887–96. 10.1128/iai.71.4.1887-1896.2003 12654805 PMC152106

[12] LeBlanc JG, Aubry C, Cortes-Perez NG, de Moreno de LeBlanc A, Vergnolle N, Langella P, Azevedo V, Chatel JM, Miyoshi A, Bermúdez-Humarán LG. Mucosal targeting of therapeutic molecules using genetically modified lactic acid bacteria: an update. FEMS Microbiol Lett. 2013; 344:1–9. 10.1111/1574-6968.12159 23600579

[13] Allain T, Mansour NM, Bahr MM, Martin R, Florent I, Langella P, Bermúdez-Humarán LG. A new lactobacilli in vivo expression system for the production and delivery of heterologous proteins at mucosal surfaces. FEMS Microbiol Lett. 2016; 363:fnw117. 10.1093/femsle/fnw117 27190148

[14] Pontes D, Innocentin S, Del Carmen S, Almeida JF, Leblanc JG, de Moreno de Leblanc A, Blugeon S, Cherbuy C, Lefèvre F, Azevedo V, Miyoshi A, Langella P, Chatel JM. Production of fibronectin binding protein a at the surface of lactococcus lactis increases plasmid transfer in vitro and in vivo. PLoS One. 2012; 7:e44892. 10.1371/journal.pone.0044892 23028664 PMC3459934

[15] Liu H, Dai C, Fan Y, Guo B, Ren K, Sun T, Wang W. From autophagy to mitophagy: the roles of P62 in neurodegenerative diseases. J Bioenerg Biomembr. 2017; 49:413–22. 10.1007/s10863-017-9727-7 28975445

[16] Cecarini V, Bonfili L, Cuccioloni M, Mozzicafreddo M, Angeletti M, Keller JN, Eleuteri AM. The fine-tuning of proteolytic pathways in Alzheimer’s disease. Cell Mol Life Sci. 2016; 73:3433–51. 10.1007/s00018-016-2238-6 27120560 PMC11108445

[17] Kuusisto E, Salminen A, Alafuzoff I. Early accumulation of p62 in neurofibrillary tangles in Alzheimer’s disease: possible role in tangle formation. Neuropathol Appl Neurobiol. 2002; 28:228–37. 10.1046/j.1365-2990.2002.00394.x 12060347

[18] Du Y, Wooten MC, Gearing M, Wooten MW. Age-associated oxidative damage to the p62 promoter: implications for Alzheimer disease. Free Radic Biol Med. 2009; 46:492–501. 10.1016/j.freeradbiomed.2008.11.003 19071211 PMC2684672

[19] Du Y, Wooten MC, Wooten MW. Oxidative damage to the promoter region of SQSTM1/p62 is common to neurodegenerative disease. Neurobiol Dis. 2009; 35:302–10. 10.1016/j.nbd.2009.05.015 19481605 PMC2718328

[20] Caccamo A, Ferreira E, Branca C, Oddo S. P62 improves AD-like pathology by increasing autophagy. Mol Psychiatry. 2017; 22:865–73. 10.1038/mp.2016.139 27573878 PMC5479312

[21] Liu WJ, Ye L, Huang WF, Guo LJ, Xu ZG, Wu HL, Yang C, Liu HF. P62 links the autophagy pathway and the ubiqutin-proteasome system upon ubiquitinated protein degradation. Cell Mol Biol Lett. 2016; 21:29. 10.1186/s11658-016-0031-z 28536631 PMC5415757

[22] Stoka V, Turk V, Turk B. Lysosomal cathepsins and their regulation in aging and neurodegeneration. Ageing Res Rev. 2016; 32:22–37. 10.1016/j.arr.2016.04.010 27125852

[23] Hook G, Yu J, Toneff T, Kindy M, Hook V. Brain pyroglutamate amyloid-β is produced by cathepsin B and is reduced by the cysteine protease inhibitor E64d, representing a potential Alzheimer’s disease therapeutic. J Alzheimers Dis. 2014; 41:129–49. 10.3233/JAD-131370 24595198 PMC4059604

[24] Hook V, Toneff T, Bogyo M, Greenbaum D, Medzihradszky KF, Neveu J, Lane W, Hook G, Reisine T. Inhibition of cathepsin B reduces beta-amyloid production in regulated secretory vesicles of neuronal chromaffin cells: evidence for cathepsin B as a candidate beta-secretase of Alzheimer’s disease. Biol Chem. 2005; 386:931–40. 10.1515/BC.2005.108 16164418

[25] Hook V, Funkelstein L, Wegrzyn J, Bark S, Kindy M, Hook G. Cysteine cathepsins in the secretory vesicle produce active peptides: cathepsin L generates peptide neurotransmitters and cathepsin B produces beta-amyloid of Alzheimer’s disease. Biochim Biophys Acta. 2012; 1824:89–104. 10.1016/j.bbapap.2011.08.015 21925292 PMC3232284

[26] Tönnies E, Trushina E. Oxidative stress, synaptic dysfunction, and Alzheimer’s disease. J Alzheimers Dis. 2017; 57:1105–21. 10.3233/JAD-161088 28059794 PMC5409043

[27] Ichimura Y, Komatsu M. Activation of p62/SQSTM1-Keap1-nuclear factor erythroid 2-related factor 2 pathway in cancer. Front Oncol. 2018; 8:210. 10.3389/fonc.2018.00210 29930914 PMC5999793

[28] Jacob KD, Noren Hooten N, Tadokoro T, Lohani A, Barnes J, Evans MK. Alzheimer’s disease-associated polymorphisms in human OGG1 alter catalytic activity and sensitize cells to DNA damage. Free Radic Biol Med. 2013; 63:115–25. 10.1016/j.freeradbiomed.2013.05.010 23684897 PMC3767440

[29] Bermúdez-Humarán LG, Aubry C, Motta JP, Deraison C, Steidler L, Vergnolle N, Chatel JM, Langella P. Engineering lactococci and lactobacilli for human health. Curr Opin Microbiol. 2013; 16:278–83. 10.1016/j.mib.2013.06.002 23850097

[30] Salminen A, Kaarniranta K, Haapasalo A, Hiltunen M, Soininen H, Alafuzoff I. Emerging role of p62/sequestosome-1 in the pathogenesis of Alzheimer’s disease. Prog Neurobiol. 2012; 96:87–95. 10.1016/j.pneurobio.2011.11.005 22138392

[31] Sabbieti MG, Agas D, Capitani M, Marchetti L, Concetti A, Vullo C, Catone G, Gabai V, Shifrin V, Sherman MY, Shneider A, Venanzi FM. Plasmid DNA-coding p62 as a bone effective anti-inflammatory/anabolic agent. Oncotarget. 2015; 6:3590–99. 10.18632/oncotarget.2884 25668818 PMC4414139

[32] Kolosova NG, Kozhevnikova OS, Telegina DV, Fursova AZ, Stefanova NA, Muraleva NA, Venanzi F, Sherman MY, Kolesnikov SI, Sufianov AA, Gabai VL, Shneider AM. P62 /SQSTM1 coding plasmid prevents age related macular degeneration in a rat model. Aging (Albany NY). 2018; 10:2136–47. 10.18632/aging.101537 30153656 PMC6128417

[33] Mancha-Agresti P, Drumond MM, Carmo FL, Santos MM, Santos JS, Venanzi F, Chatel JM, Leclercq SY, Azevedo V. A new broad range plasmid for DNA delivery in eukaryotic cells using lactic acid bacteria: in vitro and in vivo assays. Mol Ther Methods Clin Dev. 2016; 4:83–91. 10.1016/j.omtm.2016.12.005 28344994 PMC5363290

[34] Sisó S, González L, Jeffrey M. Neuroinvasion in prion diseases: the roles of ascending neural infection and blood dissemination. Interdiscip Perspect Infect Dis. 2010; 2010:747892. 10.1155/2010/747892 20652006 PMC2905956

[35] Andréoletti O, Berthon P, Marc D, Sarradin P, Grosclaude J, van Keulen L, Schelcher F, Elsen JM, Lantier F. Early accumulation of PrP^sc^ in gut-associated lymphoid and nervous tissues of susceptible sheep from a romanov flock with natural scrapie. J Gen Virol. 2000; 81:3115–26. 10.1099/0022-1317-81-12-3115 11086143

[36] Jeffrey M, Martin S, Thomson JR, Dingwall WS, Begara-McGorum I, González L. Onset and distribution of tissue prp accumulation in scrapie-affected suffolk sheep as demonstrated by sequential necropsies and tonsillar biopsies. J Comp Pathol. 2001; 125:48–57. 10.1053/jcpa.2001.0476 11437516

[37] Jeffrey M, Ryder S, Martin S, Hawkins SA, Terry L, Berthelin-Baker C, Bellworthy SJ. Oral inoculation of sheep with the agent of bovine spongiform encephalopathy (BSE). 1. Onset and distribution of disease-specific PrP accumulation in brain and viscera. J Comp Pathol. 2001; 124:280–89. 10.1053/jcpa.2001.0465 11437504

[38] Bonfili L, Cecarini V, Cuccioloni M, Angeletti M, Flati V, Corsetti G, Pasini E, Dioguardi FS, Eleuteri AM. Essential amino acid mixtures drive cancer cells to apoptosis through proteasome inhibition and autophagy activation. FEBS J. 2017; 284:1726–37. 10.1111/febs.14081 28391610

[39] Bonfili L, Cuccioloni M, Cecarini V, Mozzicafreddo M, Palermo FA, Cocci P, Angeletti M, Eleuteri AM. Ghrelin induces apoptosis in colon adenocarcinoma cells via proteasome inhibition and autophagy induction. Apoptosis. 2013; 18:1188–200. 10.1007/s10495-013-0856-0 23632965

[40] Cecarini V, Bonfili L, Cuccioloni M, Mozzicafreddo M, Rossi G, Buizza L, Uberti D, Angeletti M, Eleuteri AM. Crosstalk between the ubiquitin-proteasome system and autophagy in a human cellular model of Alzheimer’s disease. Biochim Biophys Acta. 2012; 1822:1741–51. 10.1016/j.bbadis.2012.07.015 22867901

[41] Cecarini V, Bonfili L, Cuccioloni M, Mozzicafreddo M, Rossi G, Keller JN, Angeletti M, Eleuteri AM. Wild type and mutant amyloid precursor proteins influence downstream effects of proteasome and autophagy inhibition. Biochim Biophys Acta. 2014; 1842:127–34. 10.1016/j.bbadis.2013.11.002 24215712

[42] Wei Y, Zhou J, Wu J, Huang J. Correction: ERβ promotes Aβ degradation via the modulation of autophagy. Cell Death Dis. 2019; 10:634. 10.1038/s41419-019-1867-8 31444320 PMC6707961

[43] Zhou F, van Laar T, Huang H, Zhang L. APP and APLP1 are degraded through autophagy in response to proteasome inhibition in neuronal cells. Protein Cell. 2011; 2:377–83. 10.1007/s13238-011-1047-9 21626267 PMC4875337

[44] Cho MH, Cho K, Kang HJ, Jeon EY, Kim HS, Kwon HJ, Kim HM, Kim DH, Yoon SY. Autophagy in microglia degrades extracellular β-amyloid fibrils and regulates the NLRP3 inflammasome. Autophagy. 2014; 10:1761–75. 10.4161/auto.29647 25126727 PMC4198361

[45] Pickford F, Masliah E, Britschgi M, Lucin K, Narasimhan R, Jaeger PA, Small S, Spencer B, Rockenstein E, Levine B, Wyss-Coray T. The autophagy-related protein beclin 1 shows reduced expression in early Alzheimer disease and regulates amyloid beta accumulation in mice. J Clin Invest. 2008; 118:2190–99. 10.1172/JCI33585 18497889 PMC2391284

[46] Hung SY, Huang WP, Liou HC, Fu WM. LC3 overexpression reduces Aβ neurotoxicity through increasing α7nAchR expression and autophagic activity in neurons and mice. Neuropharmacology. 2015; 93:243–51. 10.1016/j.neuropharm.2015.02.003 25686800

[47] Klionsky DJ, Abdelmohsen K, Abe A, Abedin MJ, Abeliovich H, Acevedo Arozena A, Adachi H, Adams CM, Adams PD, Adeli K, Adhihetty PJ, Adler SG, Agam G, et al. Guidelines for the use and interpretation of assays for monitoring autophagy (3rd edition). Autophagy. 2016; 12:1–222. 10.1080/15548627.2015.1100356 26799652 PMC4835977

[48] Kinney JW, Bemiller SM, Murtishaw AS, Leisgang AM, Salazar AM, Lamb BT. Inflammation as a central mechanism in Alzheimer’s disease. Alzheimers Dement (N Y). 2018; 4:575–90. 10.1016/j.trci.2018.06.014 30406177 PMC6214864

[49] Butterfield DA. Amyloid beta-peptide (1-42)-induced oxidative stress and neurotoxicity: implications for neurodegeneration in Alzheimer’s disease brain. A review. Free Radic Res. 2002; 36:1307–13. 10.1080/1071576021000049890 12607822

[50] Butterfield DA, Lauderback CM. Lipid peroxidation and protein oxidation in Alzheimer’s disease brain: potential causes and consequences involving amyloid beta-peptide-associated free radical oxidative stress. Free Radic Biol Med. 2002; 32:1050–60. 10.1016/s0891-5849(02)00794-3 12031889

[51] Kim JY, Ozato K. The sequestosome 1/p62 attenuates cytokine gene expression in activated macrophages by inhibiting IFN regulatory factor 8 and TNF receptor-associated factor 6/NF-kappaB activity. J Immunol. 2009; 182:2131–40. 10.4049/jimmunol.0802755 19201866 PMC4151355

[52] Zou X, Feng Z, Li Y, Wang Y, Wertz K, Weber P, Fu Y, Liu J. Stimulation of GSH synthesis to prevent oxidative stress-induced apoptosis by hydroxytyrosol in human retinal pigment epithelial cells: activation of Nrf2 and JNK-p62/SQSTM1 pathways. J Nutr Biochem. 2012; 23:994–1006. 10.1016/j.jnutbio.2011.05.006 21937211

[53] Wang L, Ebrahimi KB, Chyn M, Cano M, Handa JT. Biology of p62/sequestosome-1 in age-related macular degeneration (AMD). Adv Exp Med Biol. 2016; 854:17–22. 10.1007/978-3-319-17121-0_3 26427388

[54] Tilija Pun N, Park PH. Role of p62 in the suppression of inflammatory cytokine production by adiponectin in macrophages: involvement of autophagy and p21/Nrf2 axis. Sci Rep. 2017; 7:393. 10.1038/s41598-017-00456-6 28341848 PMC5428427

[55] Butterfield DA, Boyd-Kimball D. Oxidative stress, amyloid-β peptide, and altered key molecular pathways in the pathogenesis and progression of Alzheimer’s disease. J Alzheimers Dis. 2018; 62:1345–67. 10.3233/JAD-170543 29562527 PMC5870019

[56] Hickman SE, Allison EK, El Khoury J. Microglial dysfunction and defective beta-amyloid clearance pathways in aging Alzheimer’s disease mice. J Neurosci. 2008; 28:8354–60. 10.1523/JNEUROSCI.0616-08.2008 18701698 PMC2597474

[57] Ramalho JB, Soares MB, Spiazzi CC, Bicca DF, Soares VM, Pereira JG, da Silva WP, Sehn CP, Cibin FW. In vitro probiotic and antioxidant potential of Lactococcus lactis subsp. cremoris LL95 and its effect in mice behaviour. Nutrients. 2019; 11:901. 10.3390/nu11040901 31013601 PMC6521076

[58] Luerce TD, Gomes-Santos AC, Rocha CS, Moreira TG, Cruz DN, Lemos L, Sousa AL, Pereira VB, de Azevedo M, Moraes K, Cara DC, LeBlanc JG, Azevedo V, et al. Anti-inflammatory effects of lactococcus lactis NCDO 2118 during the remission period of chemically induced colitis. Gut Pathog. 2014; 6:33. 10.1186/1757-4749-6-33 25110521 PMC4126083

[59] Athari Nik Azm S, Djazayeri A, Safa M, Azami K, Ahmadvand B, Sabbaghziarani F, Sharifzadeh M, Vafa M. Lactobacilli and bifidobacteria ameliorate memory and learning deficits and oxidative stress in β-amyloid (1-42) injected rats. Appl Physiol Nutr Metab. 2018; 43:718–26. 10.1139/apnm-2017-0648 29462572

[60] Parkar SG, Kalsbeek A, Cheeseman JF. Potential role for the gut microbiota in modulating host circadian rhythms and metabolic health. Microorganisms. 2019; 7:41. 10.3390/microorganisms7020041 30709031 PMC6406615

[61] Leclercq S, Matamoros S, Cani PD, Neyrinck AM, Jamar F, Stärkel P, Windey K, Tremaroli V, Bäckhed F, Verbeke K, de Timary P, Delzenne NM. Intestinal permeability, gut-bacterial dysbiosis, and behavioral markers of alcohol-dependence severity. Proc Natl Acad Sci USA. 2014; 111:E4485–93. 10.1073/pnas.1415174111 25288760 PMC4210345

[62] Bäuerl C, Collado MC, Diaz Cuevas A, Viña J, Pérez Martínez G. Shifts in gut microbiota composition in an APP/PSS1 transgenic mouse model of Alzheimer’s disease during lifespan. Lett Appl Microbiol. 2018; 66:464–71. 10.1111/lam.12882 29575030

[63] Kaakoush NO. Insights into the role of erysipelotrichaceae in the human host. Front Cell Infect Microbiol. 2015; 5:84. 10.3389/fcimb.2015.00084 26636046 PMC4653637

[64] Charbonneau MR, Isabella VM, Li N, Kurtz CB. Developing a new class of engineered live bacterial therapeutics to treat human diseases. Nat Commun. 2020; 11:1738. 10.1038/s41467-020-15508-1 32269218 PMC7142098

[65] Billings LM, Oddo S, Green KN, McGaugh JL, LaFerla FM. Intraneuronal abeta causes the onset of early Alzheimer’s disease-related cognitive deficits in transgenic mice. Neuron. 2005; 45:675–88. 10.1016/j.neuron.2005.01.040 15748844

[66] Oddo S, Caccamo A, Shepherd JD, Murphy MP, Golde TE, Kayed R, Metherate R, Mattson MP, Akbari Y, LaFerla FM. Triple-transgenic model of Alzheimer’s disease with plaques and tangles: intracellular abeta and synaptic dysfunction. Neuron. 2003; 39:409–21. 10.1016/s0896-6273(03)00434-3 12895417

[67] Bradford MM. A rapid and sensitive method for the quantitation of microgram quantities of protein utilizing the principle of protein-dye binding. Anal Biochem. 1976; 72:248–54. 10.1006/abio.1976.9999 942051

[68] Schindelin J, Arganda-Carreras I, Frise E, Kaynig V, Longair M, Pietzsch T, Preibisch S, Rueden C, Saalfeld S, Schmid B, Tinevez JY, White DJ, Hartenstein V, et al. Fiji: an open-source platform for biological-image analysis. Nat Methods. 2012; 9:676–82. 10.1038/nmeth.2019 22743772 PMC3855844

[69] Steele RJ, Thompson AM, Hall PA, Lane DP. The p53 tumour suppressor gene. Br J Surg. 1998; 85:1460–67. 10.1046/j.1365-2168.1998.00910.x 9823903

[70] Thorpe GH, Kricka LJ, Moseley SB, Whitehead TP. Phenols as enhancers of the chemiluminescent horseradish peroxidase-luminol-hydrogen peroxide reaction: application in luminescence-monitored enzyme immunoassays. Clin Chem. 1985; 31:1335–41. 3926345

[71] Eleuteri AM, Angeletti M, Lupidi G, Tacconi R, Bini L, Fioretti E. Isolation and characterization of bovine thymus multicatalytic proteinase complex. Protein Expr Purif. 2000; 18:160–68. 10.1006/prep.1999.1187 10686146

[72] Garcia-Mazcorro JF, Dowd SE, Poulsen J, Steiner JM, Suchodolski JS. Abundance and short-term temporal variability of fecal microbiota in healthy dogs. Microbiologyopen. 2012; 1:340–47. 10.1002/mbo3.36 23170232 PMC3496977

[73] Honneffer JB, Steiner JM, Lidbury JA, Suchodolski JS. Variation of the microbiota and metabolome along the canine gastrointestinal tract. Metabolomics. 2017; 13:26 10.1007/s11306-017-1165-3

[74] Bolyen E, Rideout JR, Dillon MR, Bokulich NA, Abnet C, Al-Ghalith GA, Alexander H, Alm EJ, Arumugam M, Asnicar F, Bai Y, Bisanz JE, Bittinger K, et al QIIME 2: Reproducible, interactive, scalable, and extensible microbiome data science. PeerJ Preprints. 2018; 6:e27295v2 10.7287/peerj.preprints.27295v2 PMC7015180 31341288

[75] Callahan BJ, McMurdie PJ, Rosen MJ, Han AW, Johnson AJ, Holmes SP. DADA2: high-resolution sample inference from illumina amplicon data. Nat Methods. 2016; 13:581–83. 10.1038/nmeth.3869 27214047 PMC4927377

[76] DeSantis TZ, Hugenholtz P, Larsen N, Rojas M, Brodie EL, Keller K, Huber T, Dalevi D, Hu P, Andersen GL. Greengenes, a chimera-checked 16S rRNA gene database and workbench compatible with ARB. Appl Environ Microbiol. 2006; 72:5069–72. 10.1128/AEM.03006-05 16820507 PMC1489311

[77] Langille MG, Zaneveld J, Caporaso JG, McDonald D, Knights D, Reyes JA, Clemente JC, Burkepile DE, Vega Thurber RL, Knight R, Beiko RG, Huttenhower C. Predictive functional profiling of microbial communities using 16S rRNA marker gene sequences. Nat Biotechnol. 2013; 31:814–21. 10.1038/nbt.2676 23975157 PMC3819121

[78] Zakrzewski M, Proietti C, Ellis JJ, Hasan S, Brion MJ, Berger B, Krause L. Calypso: a user-friendly web-server for mining and visualizing microbiome-environment interactions. Bioinformatics. 2017; 33:782–83. 10.1093/bioinformatics/btw725 28025202 PMC5408814

