# Supplementary material for: Neuroprotective effects of p62(SQSTM1)-engineered lactic acid bacteria in Alzheimer’s disease: a pre-clinical study
Source: Aging (Albany NY). 2020 Aug 28;12(16):15995–6020. doi: 10.18632/aging.103900 (PMC7485699; doi:10.18632/aging.103900)
Supplement: Supplementary Materials and Methods [file aging-12-103900-s001..pdf]

SUPPLEMENTARY MATERIALS AND METHODS

Administration of *L. lactis* MG1363 strain expressing the pExu:*egfp* vector. 8-weeks-old 3xTg-AD mice were administered with LAB containing the pExu:egfp plasmid by oral gavage (10^9 CFU dissolved in 100 µL of PBS). Animals (n=8 each group) were sacrificed 6, 12, 24, 48, 72, 168 and 216 h after bacteria administration, tissues were immediately placed into 3 M ammonium sulfate in 1 M potassium citrate buffer, rinsed several times, blotted dry, and oriented in cryomolds with OCT embedding compound (Tissue-Tek Division, Miles Laboratories, Naperville, IL). Tissues were frozen by plunging the mold into nitrogen cooled isopentane. Frozen tissue blocks were stored at -70 °C until use. Four-micron frozen sections were cut in a cryostat (- 20 °C) and mounted at room temperature on acid-cleaned glass slides. Slides were immediately immersed for 5-10 mm in - 20° C acetone and then sections were washed in PBS and immediately mounted in PBS:glycerol (1:9), and allowed to dry at room temperature in a dark camera. The same sections were then used for direct immunofluorescence evaluation using a LEICA DM 2005 microscope and a Retiga 2000R digital camera (QImaging, Surrey, BC, Canada).
