# Supplementary material for: Neuroprotective effects of p62(SQSTM1)-engineered lactic acid bacteria in Alzheimer’s disease: a pre-clinical study
Source: Aging (Albany NY). 2020 Aug 28;12(16):15995–6020. doi: 10.18632/aging.103900 (PMC7485699; doi:10.18632/aging.103900)
Supplement: Supplementary Figures [file aging-12-103900-s003..pdf]

SUPPLEMENTARY FIGURES

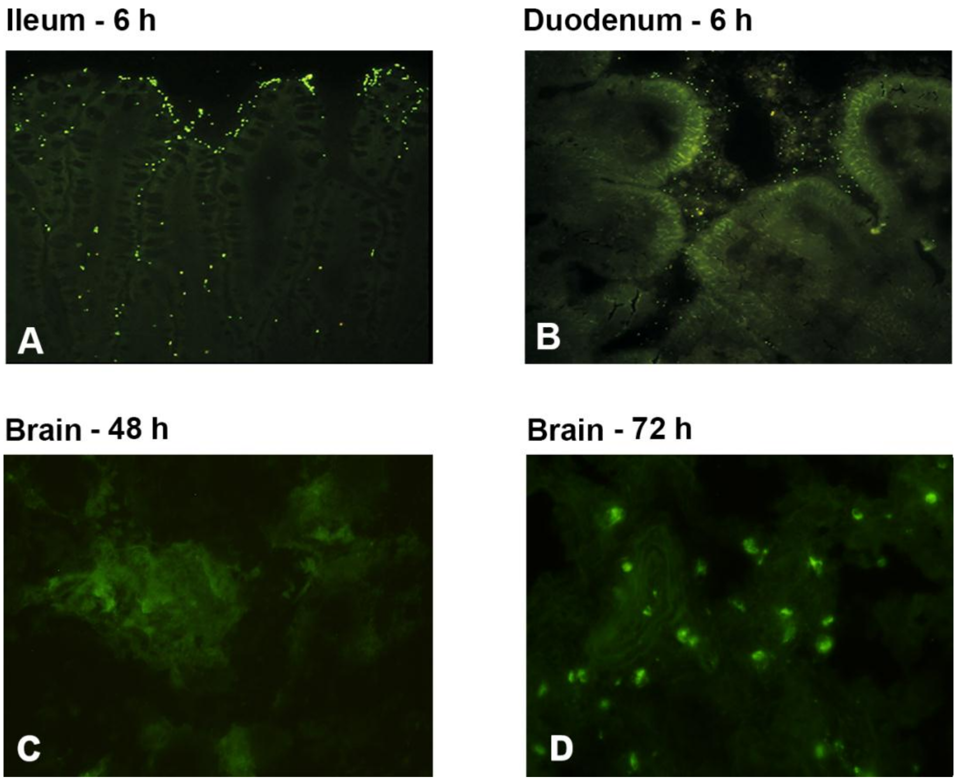

Supplementary Figure 1. eGFP expression in 3xTg-AD mice orally administered with LAB (pExu:*egfp*). Detection of eGFP in the intestine (ileum (**A**) and duodenum (**B**) portions) and brain (**C**, **D**) of 3xTg-AD mice. Fluorescence in intestinal cells was detected 6 h after the treatment and fluorescence in the brain was evidenced from 48 h to 72 h after gavage.

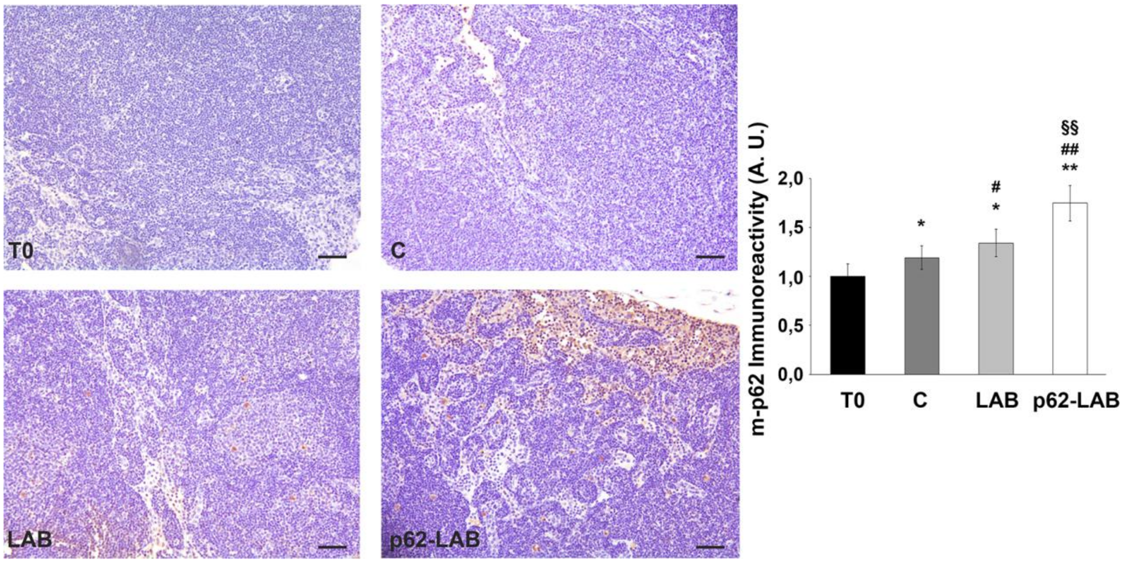

Supplementary Figure 2. Immunodetection of the m-p62 protein in mesenteric lymph nodes of 3xTg-AD mice. Mesenteric lymph nodes of p62-LAB treated 3xTg-AD mice (panel D) show a strong positivity of large groups of cells (morphologically related to mature macrophages, with some mononuclear cells). An anti-murine p62 antibody was used in the immunoassay (10×, Bar= 250μm) ($^{*}p<0.05$ and $^{**}p<0.01$ vs T0, $^{\#}p<0.05$ and $^{\#\#}p<0.01$ vs C, $^{§§}p<0.05$ vs LAB).

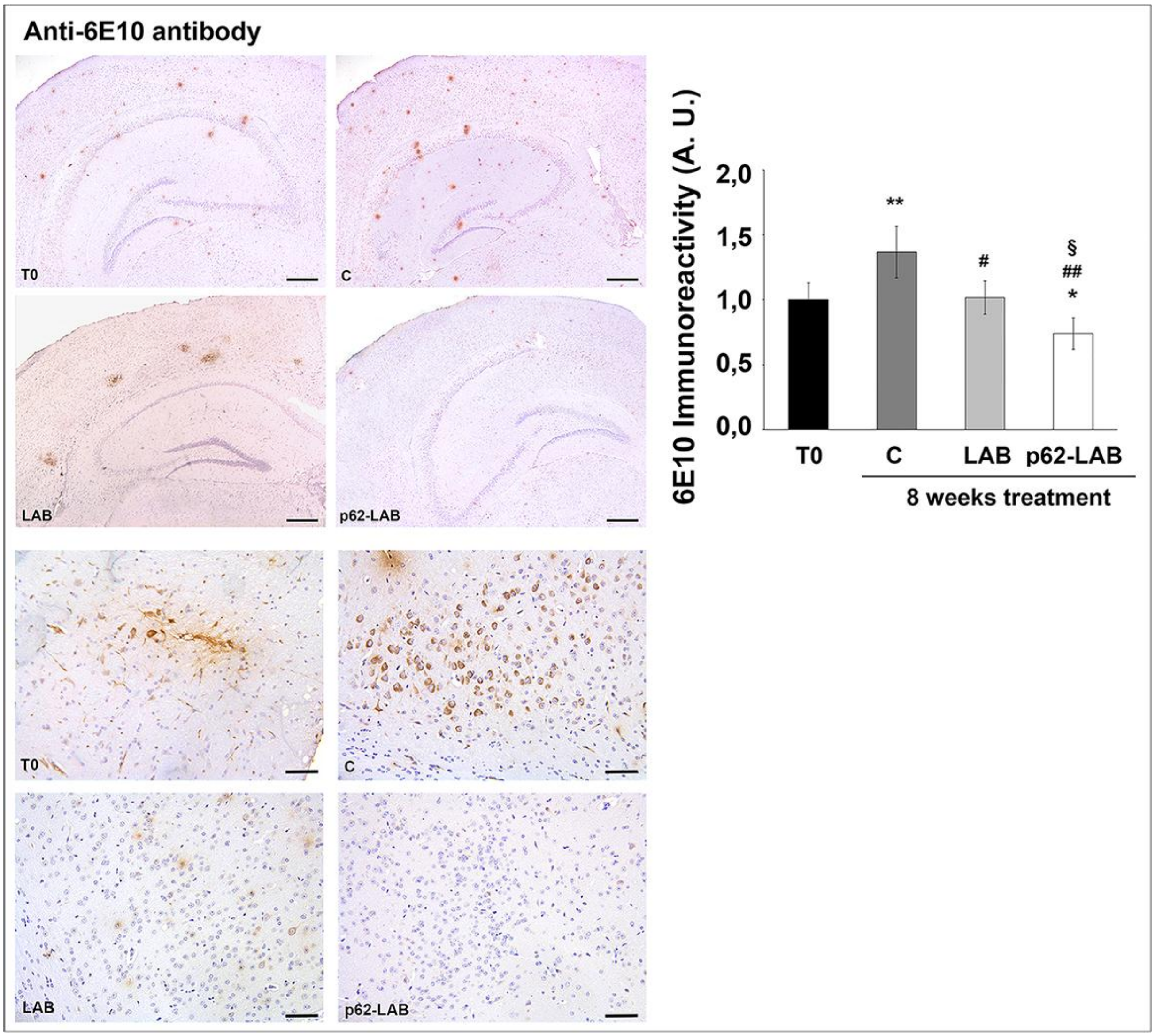

Supplementary Figure 3. Immunodetection of amyloid peptides in brain sections of 3xTg-AD mice using the anti-6E10 antibody. Immunohistochemical analysis of brain sections (magnifications 2× and 10×) from T0, C, LAB and p62-LAB groups stained with the 6E10 antibody, specific for Aβ(1–40) and Aβ(1–42) peptides and the amyloid precursor protein, and relative immunoreactivity ($^{*}p<0.05$ and $^{**}p<0.01$ vs T0, $^{\#}p<0.05$ and $^{\#\#}p<0.01$ vs C, $^{§}p<0.05$ vs LAB).

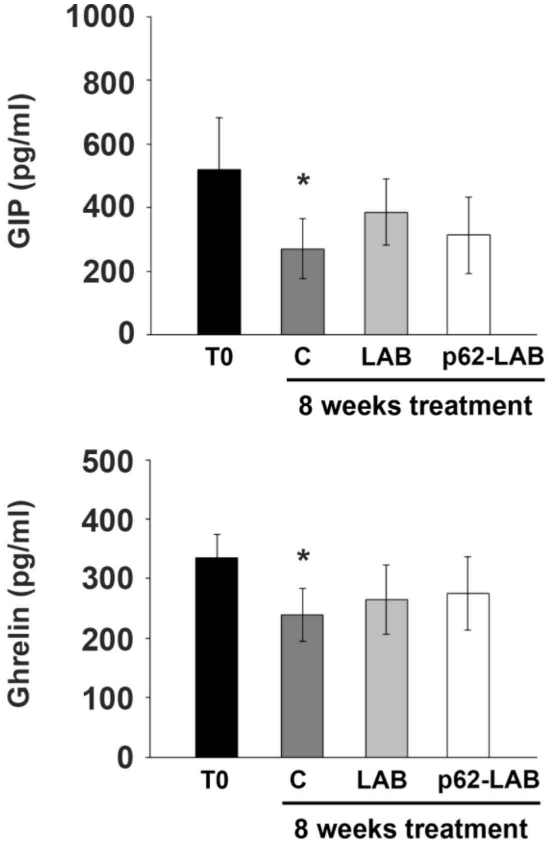

Supplementary Figure 4. Plasma concentrations of gut hormones in 3xTg-AD mice. ELISA tests were performed following the instructions of the manufacturer (see Materials and Methods for further details). Data points marked with an asterisk are statistically significant compared to untreated 8-weeks-old mice (T0, $^{*}p<0.05$).
