# Supplementary material for: Neuroprotective effects of p62(SQSTM1)-engineered lactic acid bacteria in Alzheimer’s disease: a pre-clinical study
Source: Aging (Albany NY). 2020 Aug 28;12(16):15995–6020. doi: 10.18632/aging.103900 (PMC7485699; doi:10.18632/aging.103900)
Supplement: Supplementary Table 1 [file aging-12-103900-s002..pdf]

SUPPLEMENTARY TABLE

Supplementary Table 1. KEGG pathways modified upon LAB and p62-LAB treatment. Only pathways with a p<0.05 are shown.

KEEG pathways	Groups	P-value
catechol degradation to β-ketoadipate	Control vs. p62-LAB	0.0129
3-phenylpropanoate and 3-(3-hydroxyphenyl)propanoate degradation to 2-oxopent-4-enoate	Control vs. p62-LAB	0,0295
3-phenylpropanoate and 3-(3-hydroxyphenyl)propanoate degradation to 2-oxopent-4-enoate	LAB vs. p62-LAB	0.0097
3-phenylpropanoate degradation	Control vs. p62-LAB	0.0332
3-phenylpropanoate degradation	LAB vs. p62-LAB	0.0097
toluene degradation III (aerobic) (via p-cresol)	Control vs. p62-LAB	0.0078
catechol degradation III (ortho-cleavage pathway)	Control vs. p62-LAB	0.0129
aromatic compounds degradation via β-ketoadipate	Control vs. p62-LAB	0.0129
superpathway of salicylate degradation	Control vs. p62-LAB	0.0129
4-methylcatechol degradation (ortho cleavage)	Control vs. p62-LAB	0.0129
cinnamate and 3-hydroxycinnamate degradation to 2-oxopent-4-enoate	Control vs. p62-LAB	0.0218
cinnamate and 3-hydroxycinnamate degradation to 2-oxopent-4-enoate	LAB vs. p62-LAB	0.0295
3-phenylpropanoate and 3-(3-hydroxyphenyl)propanoate degradation	Control vs. p62-LAB	0.0218
3-phenylpropanoate and 3-(3-hydroxyphenyl)propanoate degradation	LAB vs. p62-LAB	0.0295
